# Genome-wide association study in individuals of European and African ancestry and multi-trait analysis of opioid use disorder identifies 19 independent genome-wide significant risk loci

**DOI:** 10.1038/s41380-022-01709-1

**Published:** 2022-07-25

**Authors:** Joseph D. Deak, Hang Zhou, Marco Galimberti, Daniel F. Levey, Frank R. Wendt, Sandra Sanchez-Roige, Alexander S. Hatoum, Emma C. Johnson, Yaira Z. Nunez, Ditte Demontis, Anders D. Børglum, Veera M. Rajagopal, Mariela V. Jennings, Rachel L. Kember, Amy C. Justice, Howard J. Edenberg, Arpana Agrawal, Renato Polimanti, Henry R. Kranzler, Joel Gelernter

**Affiliations:** 1 Yale School of Medicine, New Haven, CT USA; 2 VA Connecticut Healthcare Center, West Haven, CT USA; 3 University of California San Diego, La Jolla, CA USA; 4 Vanderbilt University Medical Center, Nashville, TN USA; 5 Washington University St. Louis Medical School, St. Louis, MO USA; 6 Biomedicine, Aarhus University, Aarhus, Denmark; 7 Lundbeck Foundation Initiative for Integrative Psychiatric Research, iPSYCH, Aarhus, Denmark; 8 Center for Genomics and Personalized Medicine, Aarhus, Denmark; 9 University of Pennsylvania Perelman School of Medicine, Philadelphia, PA USA; 10 Crescenz VA Medical Center, Philadelphia, PA USA; 11 Indiana University School of Medicine, Indianapolis, IN USA

**Keywords:** Genetics, Addiction

## Abstract

Despite the large toll of opioid use disorder (OUD), genome-wide association studies (GWAS) of OUD to date have yielded few susceptibility loci. We performed a large-scale GWAS of OUD in individuals of European (EUR) and African (AFR) ancestry, optimizing genetic informativeness by performing MTAG (Multi-trait analysis of GWAS) with genetically correlated substance use disorders (SUDs). Meta-analysis included seven cohorts: the Million Veteran Program, Psychiatric Genomics Consortium, iPSYCH, FinnGen, Partners Biobank, BioVU, and Yale-Penn 3, resulting in a total *N* = 639,063 (*N*_cases_ = 20,686;N_effective_ = 77,026) across ancestries. OUD cases were defined as having a lifetime OUD diagnosis, and controls as anyone not known to meet OUD criteria. We estimated SNP-heritability (h^2^_SNP_) and genetic correlations (r_g_). Based on genetic correlation, we performed MTAG on OUD, alcohol use disorder (AUD), and cannabis use disorder (CanUD). A leave-one-out polygenic risk score (PRS) analysis was performed to compare OUD and OUD-MTAG PRS as predictors of OUD case status in Yale-Penn 3. The EUR meta-analysis identified three genome-wide significant (GWS; *p* ≤ 5 × 10^−^^8^) lead SNPs—one at *FURIN* (rs11372849; *p* = 9.54 × 10^−^^10^) and two *OPRM1* variants (rs1799971, *p* = 4.92 × 10^−^^09^; rs79704991, *p* = 1.11 × 10^−^^08^; r^2^ = 0.02). Rs1799971 (p = 4.91 × 10^−^^08^) and another *OPRM1* variant (rs9478500; *p* = 1.95 × 10^−^^08^; r^2^ = 0.03) were identified in the cross-ancestry meta-analysis. Estimated h^2^_SNP_ was 12.75%, with strong r_g_ with CanUD (r_g_ = 0.82; *p* = 1.14 × 10^−^^47^) and AUD (r_g_ = 0.77; *p* = 6.36 × 10^−^^78^). The OUD-MTAG resulted in a GWAS N_equivalent_ = 128,748 and 18 independent GWS loci, some mapping to genes or gene regions that have previously been associated with psychiatric or addiction phenotypes. The OUD-MTAG PRS accounted for 3.81% of OUD variance (beta = 0.61;s.e. = 0.066; *p* = 2.00 × 10^−^^16^) compared to 2.41% (beta = 0.45; s.e. = 0.058; *p* = 2.90 × 10^−^^13^) explained by the OUD PRS. The current study identified OUD variant associations at *OPRM1*, single variant associations with *FURIN*, and 18 GWS associations in the OUD-MTAG. The genetic architecture of OUD is likely influenced by both OUD-specific loci and loci shared across SUDs.

## Introduction

Opioid use disorder (OUD) has a serious negative impact on public health and is a leading cause of preventable death [1]. Although opioid misuse and progression to OUD [2] are influenced by heritable factors, discovery of OUD risk loci has been limited [3–7]. Difficulties in advancing OUD genetic discovery are largely due to lack of adequately powered cohorts of genetically informative samples [8, 9].

Genome-wide association studies (GWAS) examining single nucleotide polymorphism (SNP) effects on OUD have been underpowered [8, 9]. Nevertheless, recent progress in GWAS of OUD include the identification and confirmation of a genome-wide significant (GWS) functional variant (rs1799971) in *OPRM1* [7]. Earlier OUD GWAS identified associations with variation in several genes including *KCNG2*, *KCNC1*, *APBB2*, *CNIH3*, *RGMA*, and *OPRM1* [3–6], but the validity of those associations remains largely untested due to the lack of powerful independent OUD cohorts. OUD GWAS have also demonstrated genetic correlations (r_g_) with other substance use disorders (SUDs) (e.g. alcohol use disorder [AUD]; *r*_g_ = 0.73) and psychiatric disorders (e.g. attention-deficit hyperactivity disorder; [r_g_ = 0.36]) [7].

Large-scale GWAS meta-analyses have advanced discovery of novel loci for SUDs (e.g., AUD, problematic alcohol use (PAU), cannabis use disorder (CanUD) [10–12]. This study applies similar meta-analytic methods for OUD, combining GWAS effects across multiple studies and two ancestral groups.

Multi-trait methods (e.g., MTAG; Multi-trait analysis of GWAS) [13] have the potential to increase power. MTAG capitalizes on the r_g_ between genetically-related traits (e.g., r_g_ > 0.70) to increase the equivalent sample size. MTAG is an attractive option for boosting power for sets of similar traits like SUDs [11, 14], and holds particular promise for disorders such as OUD for which only limited cases are available for analysis. MTAG can generate estimates of trait-specific effects that leverage information from multiple GWAS summary statistics while accounting for both known and unknown sample overlap across the discovery samples [13]. MTAG can maximize the genetic information available for OUD by leveraging the statistical power of GWAS of non-opioid SUDs.

We conducted a large-scale GWAS meta-analysis of OUD in samples of African (AFR) and European (EUR) ancestry individuals. We maximized the informativeness of the available samples by performing a multi-trait analysis that incorporates SUDs that are highly genetically correlated with OUD.

## Methods

### Data and participants

The meta-analysis includes summary statistics across seven cohorts examining OUD case vs. OUD control status in AFR and EUR ancestry individuals. We included both published and unpublished OUD GWAS. Previously published GWAS include data from Yale-Penn [3, 6, 7], PGC-SUD [6], and the Partners Biobank [15]. For MVP Releases 1 and 2 (the data releases used in the present analysis), a previous GWAS of OUD cases vs. opioid-exposed controls was reported [7]. MVP data included in the current meta-analysis use a different control definition (unscreened controls) to align better with the control definitions available in most other included samples. GWAS summary data for FinnGen [16] was accessed via a publicly available repository (https://r5.finngen.fi/). GWAS of OUD from iPSYCH [17], BioVU [18], and newly-available data from Yale-Penn subjects (Yale-Penn 3), previously unpublished, were performed by analysts at their respective study sites (Supplemental Materials). We had a total AFR *N* = 84,877 (*N*_case_ = 5435 *N*_effective_ = 20,032), a total EUR *N* = 554,186 (*N*_case_ = 15,251; *N*_effective_ = 56,994), and an overall *N* = 639,063 (*N*_case_ =20,686; *N*_effective_ = 77,026). Other than Yale-Penn, this study involved de-identified data. The work was approved as appropriate by the Central Veterans Affairs (VA) institutional review board (IRB) and site-specific IRBs, including Yale University School of Medicine and VA Connecticut, and was conducted in accordance with all relevant ethical regulations. Cohort-specific summaries of AFR and EUR OUD subjects are presented in Table 1. Specific OUD diagnostic codes are provided in Supplementary Table 1. Additional phenotyping considerations are described in Supplemental Materials.

**Table 1:** Overview of samples included in GWAS meta-analysis of OUD cases vs. OUD controls.

Cohorts	EUR cases	EUR controls	EUR N_effective_	AFR cases	AFR Controls	AFR N_effective_	Case definition	Control definition^a^
MVP 1-2 combined	8529	267,737	33,062	4032	71,511	15,265	ICD-9/ICD-10	unscreened
MVP Release 1	6367	202,636	24,692	3151	54,178	11,911		
MVP Release 2	2162	65,101	8370	881	17,333	3354		
PGC-SUD^b^	3272	25,437	11,596	1231	7063	4193	DSM-IV	unexposed
Partners Biobank	1039	20,271	3953	-	-	-	ICD-9/ICD-10	no SUD diagnosis
BioVU	933	3,732	2,986	-	-	-	ICD-9/ICD-10	unscreened
FinnGen	651	214,999	2596	-	-	-	ICD-9/ICD-10	unscreened
Yale-Penn 3	448	1538	1388	172	868	574	DSM-IV	no OUD diagnosis
iPSYCH	379	5221	1413	-	-	-	ICD-9/ICD-10	no OUD diagnosis
Ancestry-specific subtotals	15251	538,935	56,994	5435	79,442	20,032		
Overall total cases	20,686							
Overall total controls	618,377							
Overall total *N*	639,063	Total N_effective_	77,026					
EUR total *N*	554,186	EUR N_effective_	56,994					
AFR total *N*	84,877	AFR N_effective_	20,032					

^a^Control definition: “no OUD diagnosis”—controls assessed for OUD and not diagnosed. “Unscreened”—controls not assessed for OUD and thus may have unassessed opioid-related problems.^b^The PGC-SUD OUD analysis included AFR and EUR participants from Yale-Penn 1 (*N* = 3922; Ncases = 1656) and Yale-Penn 2 (*N* = 2483; Ncases = 846)—data from Yale-Penn supplied 67.4% of AFR cases and 51.1% of EUR cases for the overall PGC-SUD meta-analysis. Yale-Penn 3 is included as a separate cohort in the current analysis. Effective sample size was calculated as: N_effective_ = 4/[(1/n_case_) + (1/n_control_)].

### Ancestry-specific and cross-ancestry GWAS meta-analysis

**Fig. 1:**
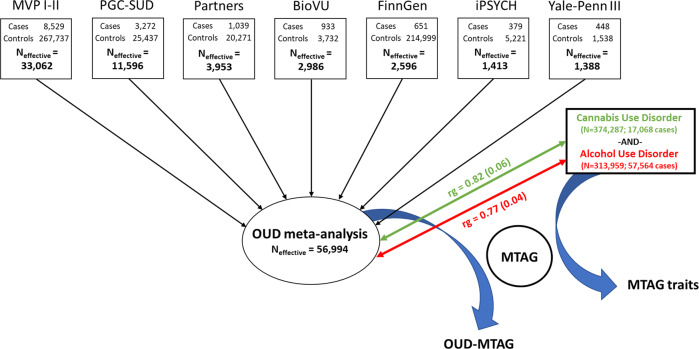
Summary of OUD GWAS, meta-analysis, and MTAG study design. Overview of European-ancestry opioid use disorder (OUD) genome-wide association study and OUD multi-trait analysis.

GWAS samples were combined using an effective sample-size weighted meta-analysis in METAL[19]. Ancestry-specific and cross-ancestry meta-analyses were performed. Measures of cross-sample heterogeneity (Cochran’s Q, I^2^) and genomic inflation (λ_GC_) were used to evaluate potential bias influenced by heterogeneity between cohorts or by population stratification. Included GWAS summary statistics were limited to variants present in at least 80% of the analysis-specific effective sample size (e.g., 80% of EUR N_effective_). The 80% effective sample size inclusion threshold ensured that variant effects present only in smaller cohorts did not disproportionately influence the overall results. This effectively meant that a variant needed to be present in MVP, PGC-SUD, and at least one additional cohort for it to be included (Fig. 1).

Data from the 1000 Genome Project (1000 G) phase 3 [20] was used for LD reference. Variants were mapped to the nearest gene based upon physical position (<10 kb from assigned gene) and further characterized by gene-mapping approaches using expression quantitative trait locus (eQTL) associations and 3D chromatin interactions (Hi-C)(Supplemental Materials). Conditional analyses were conducted using GCTA-COJO [21] to examine the conditional independence of genome-wide significant (GWS; *p* = 5.00 × 10^−^^08^) *OPRM1* variants in low LD (r^2^ < 0.1).

### SNP-heritability and Linkage-Disequilibrium (LD) Score Regression

EUR OUD GWAS summary statistics were used to estimate SNP-heritability (h^2^_SNP_), and to characterize OUD genetic correlations (r_g_) using LD score regression (LDSC) [22]. LDSC analyses were restricted to HapMap3 variants [23]. Effective sample-size was used in all LDSC-based analytic steps. Genetic correlations were estimated with 54 other traits including SUDs, substance use, psychiatric traits, chronic pain, sociodemographic factors, and additional traits of interest (data sources are described in Supplemental Tables). Bonferroni-corrected significance was *p* = 9.26 × 10^−04^ (0.05/54). LDSC analyses were not performed in AFR and cross-ancestry meta-analyses because of the inability to use an LD reference panel for recently-admixed populations such as African-Americans or for analyses integrating datasets from diverse ancestry groups [22].

### Multi-trait analysis of GWAS summary statistics (MTAG)

Based on LDSC estimates of genetic correlations with OUD, a joint-analysis that included the EUR OUD GWAS and GWAS summary statistics for AUD [11] and CanUD [12] was conducted using MTAG [13]. MTAG enhances statistical power by leveraging the genetic correlation between traits to generate trait-specific estimates for each SNP. The AUD GWAS summary statistics used in the present analysis are from a broader GWAS of problematic alcohol use [11]. MTAG used study-specific effective sample sizes for the respective GWAS. Study-specific effect sizes were transformed to Z-scores to be on a uniform scale across the three included GWAS. Included genetic variants were filtered using default MTAG parameters [13]. Briefly, variants were restricted to those common to all three of the GWAS, with a minor allele frequency (MAF) > 0.01, and present in at least two-thirds of the 90th percentile of the study-specific SNP sample sizes. These MTAG parameters guard against heterogeneity in the distribution of common vs. rare variant effects, ensuring that SNP effects generated from relatively small subsets of the contributing discovery GWAS do not bias the effect estimates across traits [13].

### Phenome-wide Association Study (PheWAS)

To examine phenome-wide relationships for OUD and the OUD-MTAG analysis, and to compare their relationships with other clinically-relevant outcomes, we performed phenome-wide association studies (PheWAS) in BioVU [18], a cohort of >66,000 genotyped patients, with phenotypic data currently available for 1338 clinical outcomes from electronic health records [18]. Additional details on the BioVU cohort are provided in Supplemental Materials. Polygenic risk scores (PRS) for OUD and OUD-MTAG were computed using PRS-CS [24], excluding the subset of BioVU participants included in the meta-analysis. The respective PRS were then included in individual logistic regression models regressed on 1291 clinical outcomes with case counts ≥100, covarying for sex, age, and the first 10 genetic principal components. Statistical significance for the PheWAS was defined as *p* = 3.87 × 10^−^^05^ (0.05/1291).

### Polygenic risk score analysis

PRS are described in Supplemental Materials. Briefly, a leave-one-out PRS analysis was performed by excluding the EUR and AFR Yale-Penn 3 (YP3) cohorts from the respective OUD GWAS and OUD-MTAG analyses allowing for YP3 OUD cases and controls to be used as ancestry-specific PRS target samples.

## Results

### Ancestry-specific and cross-ancestry GWAS meta-analyses

**Fig. 2:**
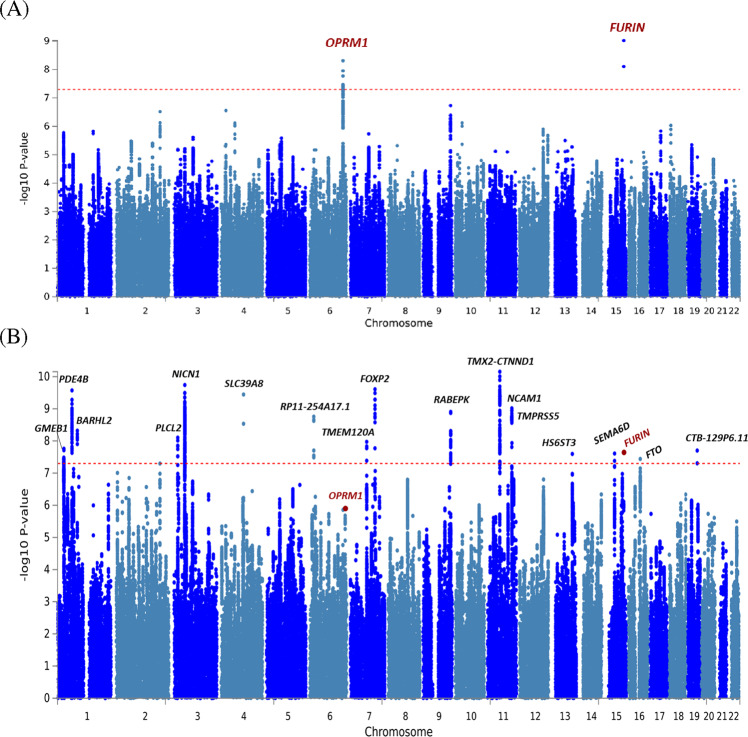
OUD and OUD-MTAG manhattan plots. Manhattan plots of (**A**) European-ancestry OUD GWAS results and (**B**) OUD-MTAG multi-trait GWAS results.

In the ancestry-specific analyses, there were three GWS variants (Fig. 2) in EUR (Table 2). The top association (rs11372849; *p* = 9.54 × 10^−^^10^) mapped to *FURIN* on chromosome 15, one of two GWS SNPs in the *FURIN* gene (rs17514846; r^2^ = 0.91). The second strongest association was with the *OPRM1* functional variant (rs1799971; *p* = 4.92 × 10^−^^09^). An additional *OPRM1* variant was also identified (rs79704991; *p* = 1.11 × 10^−^^08^; r^2^ = 0.02)(*OPRM1* regional plots—Supplementary Fig. 1). GCTA-COJO [21] was used for conditional analysis of the two GWS *OPRM1* variants demonstrating low LD (rs1799971 conditioned on rs79704991 and vice versa). In these analyses, each variant fell below GWS when conditioning on the effect of the other (conditioned rs1799971-*p*_*conditioned*_ = 1.66 × 10^−^^06^; rs79704991-*p*_*conditioned*_ = 3.71 × 10^−^^06^); although, there were no statistically significant differences in effect estimates for the respective *OPRM1* variants conditioned vs. unconditioned effects. No GWS variants were identified in the AFR GWAS (Supplementary Fig. 2).

The cross-ancestry OUD GWAS identified two GWS risk variants mapping to *OPRM1* (Supplementary Fig. 3) (Table 2). The top association was with rs9478500 (*p* = 1.95 × 10^−^^08^), an intronic variant. Rs1799971 was also GWS in the cross-ancestry meta-analysis (*p* = 4.91 × 10^−^^08^), and is not in strong LD with rs9478500 (EUR r^2^ = 0.03; AFR r^2^ = 0.002; ALL r^2^ = 0.04). The top *FURIN* association in EUR (rs11372849) was uninformative in three of four AFR ancestry cohorts and did not meet the threshold (80% of N_effective_) we set to be included in the analysis. The second top *FURIN* association (rs17514846) fell below GWS in the cross-ancestry GWAS (*p* = 6.00 × 10^−^^08^).

**Table 2:** Genome-wide significant (*p* ≤ 5.00E-08) associations in (A) EUR OUD GWAS, and (B) cross-ancestry OUD GWAS.

(A) EUR OUD analysis														
Chr	Position	Marker	A1	A2	Gene	EUR MAF	EUR Z	EUR P-value	Direction	AFR MAF	AFR Z	AFR *P*-value		
15	91419432	**rs11372849**	T	TC	*FURIN*	0.46	6.12	9.54E-10	+ + + ??? + ?	-	-	-		
6	154360797	**rs1799971**	A	G	*OPRM1*	0.13	5.85	4.92E-09	+ + + + + + + +	0.03	0.83	0.407		
15	91416550	rs17514846	A	C	*FURIN*	0.46	−5.77	7.87E-09	---- + --?	0.18	−0.94	0.347		
6	154319449	**rs79704991**	T	G	*OPRM1*	0.13	5.71	1.11E-08	+ + + + + + + +	0.08	0.59	0.552		
6	154315310	rs12200046	T	C	*OPRM1*	0.13	5.64	1.70E-08	+ + + + + + + +	0.08	0.67	0.505		
6	154309808	rs10499276	T	C	*OPRM1*	0.13	5.52	3.38E-08	+ + + + + + + +	0.08	0.49	0.621		
6	154304242	rs34069531	T	C	*OPRM1*	0.13	5.51	3.67E-08	+ + + + + + + +	0.08	0.61	0.542		
6	154377925	rs3778146	T	C	*OPRM1*	0.17	−5.5	3.84E-08	--- + ----	0.08	−1.54	0.125		
6	154378223	rs9478500	T	C	*OPRM1*	0.17	−5.49	4.12E-08	--- + ----	0.08	−1.76	0.078		
6	154379152	rs3823010	A	G	*OPRM1*	0.17	5.48	4.25E-08	+ + + - + + + +	0.08	1.59	0.111		
6	154378739	rs3778147	A	G	*OPRM1*	0.17	5.46	4.83E-08	+ + + - + + + +	0.08	1.47	0.141		

(B) Cross-ancestry OUD analysis														
Chr	Position	Marker	A1	A2	Gene	Z	*P*-value	Direction	EUR MAF	EUR Z	EUR *P*-value	AFR MAF	AFR Z	AFR *P*-value
6	154378223	**rs9478500**	T	C	*OPRM1*	−5.62	1.95E-08	--- + ----- + - +	0.17	−5.49	4.12E-08	0.18	−1.76	0.078
6	154379934	rs9285542	T	C	*OPRM1*	5.56	2.73E-08	+ + + - + + + + + - + +	0.17	5.43	5.61E-08	0.16	1.74	0.082
6	154379152	rs3823010	A	G	*OPRM1*	5.53	3.26E-08	+ + + - + + + + + + --	0.17	5.48	4.25E-08	0.11	1.59	0.111
6	154381012	rs3778148	T	G	*OPRM1*	5.52	3.34E-08	+ + + - + + + + + + - +	0.17	5.43	5.73E-08	0.10	1.68	0.094
6	154355100	rs6936615	A	G	*OPRM1*	−5.52	3.38E-08	--- + --?--- + -	-	-	-	0.10	−1.72	0.085
6	154377925	rs3778146	T	C	*OPRM1*	−5.51	3.54E-08	--- + ------ + +	0.17	−5.5	3.84E-08	0.12	−1.54	0.125
6	154383658	rs3778150	T	C	*OPRM1*	−5.49	3.97E-08	--- + ----- + - +	0.17	-5.38	7.31E-08	0.18	−1.69	0.091
6	154382139	rs3778149	C	G	*OPRM1*	−5.48	4.20E-08	--- + ------ + +	0.17	−5.44	5.42E-08	0.12	−1.58	0.114
6	154382473	rs7772959	A	G	*OPRM1*	5.48	4.34E-08	+ + + - + + + + + + --	0.17	5.43	5.66E-08	0.12	1.58	0.114
6	154362254	rs9322445	A	G	*OPRM1*	−5.47	4.54E-08	--- + ------ + -	0.17	−5.44	5.49E-08	0.10	−1.56	0.120
6	154382367	rs7773995	T	C	*OPRM1*	5.46	4.78E-08	+ + + - + + + + + + --	0.17	5.42	5.84E-08	0.12	1.56	0.120
6	154360797	**rs1799971**	A	G	*OPRM1*	5.46	4.91E-08	+ + + + + + + + + - + +	0.13	5.85	4.92E-09	0.03	0.83	0.407
Bold = Lead SNP														

### Gene-based analysis

Gene-based analyses are described in Supplemental Materials. Both *FURIN* (*p* = 3.09 × 10^−^^07^) and *OPRM1* (*p* = 3.59 × 10^−^^07^) were significant in EUR gene-based analysis (Supplementary Fig 4). Additional results are reported in Supplemental Table 3–5 and Supplementary Fig 5.

### SNP-heritability and Linkage-Disequilibrium (LD) Score Regression

The liability scale SNP-heritability (h^2^_SNP_) estimate was 12.75% (s.e. = 1.1%) in EUR using effective sample-size adjusted prevalence rates and a population prevalence of 0.021 [25]. Genome-wide inflation was mild with respect to sample size and favored OUD polygenicity as indicated by the LDSC inflation factor (λ_GC_ = 1.18), intercept = 1.01 (s.e. = 0.011), and attenuation ratio = 0.05 (s.e. = 0.049).

**Fig. 3:**
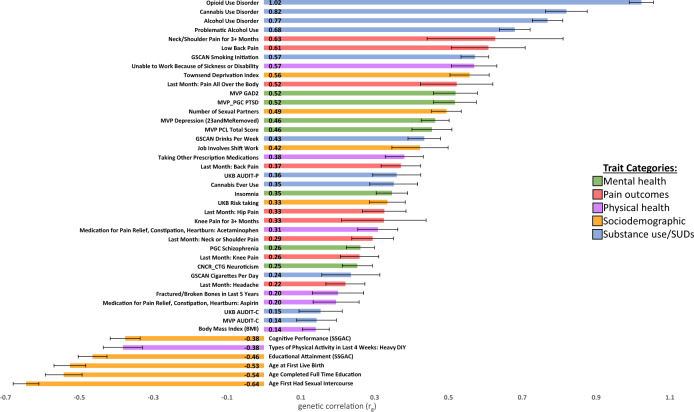
OUD genetic correlation results. EUR OUD GWAS genetic correlations (r_g_) with mental health, pain, physical health, sociodemographic, and substance use traits of interest.

OUD showed statistically significant (*p* ≤ 9.26 x 10^−0^^4^) genetic correlations with 40 traits including substance use, SUDs, psychiatric traits, pain outcomes, physical health, and sociodemographic characteristics (Fig. 3**;** Supplementary Table 6). The OUD trait in the current study was strongly genetically correlated with the largest published GWAS of OUD to date (r_g_ = 1.02; *p* = 2.38 x 10^−^^214^) [7], suggesting that OUD is being captured consistently across the studies, as might be expected given the substantial overlap in OUD cases between the two studies, although the control definitions differed between analyses. OUD was also strongly genetically correlated with other SUDs, including CanUD (r_g_ = 0.82; *p* = 1.14 x 10^−^^47^) [12] and AUD (r_g_ = 0.77; *p* = 6.36 x 10^−^^78^) [11]. Modest genetic correlations were found for measures of substance use (e.g., the quantity/frequency alcohol use measure AUDIT-C) (r_g_ = 0.14; *p* = 8.15 x 10^−^^03^) [10].

OUD also demonstrated statistically significant genetic correlations with many mental health, pain, physical health, and sociodemographic traits. The strongest positive correlations across the respective domains were with Generalized Anxiety Disorder (r_g_ = 0.52; *p* = 2.89 x 10^−^^18^) and PTSD (r_g_ = 0.52; *p* = 3.87 x 10^−^^19^), lower back pain (r_g_ = 0.61; *p* = 1.22 x 10^−^^09^), inability to work due to being sick or disabled (r_g_ = 0.57; *p* = 1.31 x 10^−^^20^), and scores on the Townsend Deprivation Index (r_g_ = 0.56; *p* = 1.13 x 10^−^^25^). OUD was negatively genetically correlated with measures of sexual activity (age of first sexual intercourse [r_g_ = −0.64; *p* = 4.43 x 10^−^^76^]), indices of educational attainment (age of school completion [r_g_ = −0.54; *p* = 9.41x10^−^^28^]) and cognitive performance (r_g_ = −0.38; *p* = 1.54 x 10^−^^20^), and levels of past month “Heavy Do It Yourself” physical activity (r_g_ = −0.38; *p* = 7.37 x 10^−^^13^), among others (Supplementary Table 6).

### Multi-trait analysis of European GWAS summary statistics (MTAG)

MTAG was supported by strong genetic correlation for OUD with CanUD (r_g_=0.82; *p* = 1.14 x 10^−^^47^) and AUD (r_g_ = 0.77; *p* = 6.36 x 10^−^^78^) in EUR. The OUD-MTAG analysis resulted in an increase in effective sample size from the original EUR OUD GWAS N_effective_ = 56,994 (GWAS mean *x*^2^ = 1.18) to an equivalent sample size of *N* = 128,748 (GWAS mean *x*^2^ = 1.40). The increase resulted in the identification of 18 independent GWS OUD-MTAG risk loci (Fig. 2**;** Table 3), some previously associated at either the variant level, or that reside in genes associated with, psychiatric and substance use outcomes in previous GWAS. Seven of the OUD-MTAG loci mapped to the nearest gene via brain eQTL data and Hi-C interactions; 8 loci were not mapped to the nearest gene via brain eQTL and Hi-C data but were implicated with additional genes in their respective genomic regions. Some loci fell in complex genomic regions with many mapped genes. These OUD-MTAG loci regions are summarized in Supplemental Fig. 6, 7 and Supplementary Tables 12–15 along with the OUD loci.

**Table 3:** Genome-wide significant (*p* ≤ 5.00 × 10^−^^08^) Lead SNP associations in OUD-MTAG analysis (of 441 total GWS SNPs).

Chr	Pos	MarkerName	Allele 1	Allele 2		Gene(s)	Z	*P*-value	*p*-value in AUD	*p*-value in CanUD
11	57535966	rs11229119	T		C	*TMX2-CTNND1*	−6.50	7.03E-11	2.60E-07	6.68E-05
3	49469449	rs77648866	A		G	*NICN1*	6.33	1.82E-10	5.90E-07	5.89E-03
7	114137940	rs1989903	A		G	*FOXP2*	−6.20	2.47E-10	1.23E-03	**3.52E-09**
1	66434743	rs7519259	A		G	*PDE4B*	5.73	2.68E-10	9.94E-08	1.36E-05
4	103198082	rs13135092	A		G	*SLC39A8*	6.51	3.60E-10	**4.89E-18**	0.88
11	112869404	rs1940701	T		C	*NCAM1*	-6.10	9.63E-10	4.91E-06	1.67E-03
9	127968109	rs864882	T		C	*RABEPK*	−5.60	1.24E-09	2.45E-05	0.06
6	19076417	rs9350100	T		C	*RP11-254A17.1*	5.62	1.76E-09	3.37E-05	2.89E-03
1	91208451	rs2166171	T		C	*BARHL2*	−5.88	4.80E-09	1.18E-06	2.76E-05
3	16850764	rs55855024	A		C	*PLCL2*	5.73	7.89E-09	1.50E-06	0.20
7	75622281	rs6467958	T		C	*TMEM120A*	−5.50	1.06E-08	2.24E-05	4.02E-03
11	113477081	rs11214677	T		C	*TMPRSS5*	−5.65	1.16E-08	1.23E-04	1.65E-05
1	28989020	rs6667501	A		G	*GMEB1*	5.55	1.74E-08	3.66E-04	2.21E-03
19	45453763	rs10422888	A		G	*CTB-129P6.11*	5.55	1.99E-08	5.83E-05	6.22E-03
15	91416550	rs17514846	A		C	*FURIN*	−5.59	2.30E-08	2.85E-03	0.09
15	47645174	rs73403005	A		G	*SEMA6D*	−5.69	2.46E-08	**6.06E-09**	2.38E-03
13	96932868	rs2389631	A		C	*HS6ST3*	−5.61	2.53E-08	8.72E-05	0.06
16	53834607	rs7188250	T		C	*FTO*	5.64	3.63E-08	**4.41E-12**	0.61
									Bold = GWS in non-OUD MTAG sumstats	

The top OUD-MTAG association was with rs11229119 (*p* = 7.03 x 10^−^^11^) on chromosome 11 mapping to both *TMX2* and *CTNND1*. The second strongest was with *NICN1* (rs77648866; *p* = 1.82 x 10^−^^10^) on chromosome 3. Additional GWS associations included *FOXP2* (rs1989903; *p* = 2.47 x 10^−^^10^), *PDE4B* (rs7519259; *p* = 2.68 x 10^−^^10^), *SLC39A8* (rs13135092; *p* = 3.60 x 10^−^^10^), *NCAM1* (rs1940701; *p* = 9.63 x 10^−^^10^), *RABEPK* (rs864882; *p* = 1.24 x 10^−^^09^), *PLCL2* (rs55855024; *p* = 7.89 x 10^−^^09^), and *FTO* (rs7188250; *p* = 3.63 x 10^−^^08^). One of the *FURIN* variants identified in the EUR OUD GWAS was also GWS in the OUD-MTAG (rs17514846; *p* = 2.30 x 10^−^^08^). The top *OPRM1* association was with rs1799971 (*p* = 1.39 x 10^−^^06^). Of the 18 GWS loci, three were GWS in the AUD GWAS and one was GWS in the CanUD GWAS used for MTAG **(**Table 3**)**.

The OUD-MTAG gene-based analysis resulted in 66 Bonferroni significant (*p* ≤ 0.05/15,927 = 3.14 x 10^−0^^6^) genes (Supplementary Table 7**;** Supplementary Fig 8).

The OUD-MTAG GWAS was significantly genetically correlated (Bonferroni *p* ≤ 9.26 x 10^−0^^4^) with 46 traits including the largest previously published GWAS of OUD to date at r_g_ = 0.98 (*p* = 1.22 x 10^−^^77^) [7]. All estimates of genetic correlation for the OUD-MTAG analysis can be found in Supplementary Table 8.

### Phenome-wide Association Study (PheWAS)

The top PheWAS association for OUD was with substance addiction and disorders (OR = 1.53; *p* = 2.12 x 10^−^^69^). Additional top OUD associations included tobacco use disorder (OR = 1.26; *p* = 3.38 x 10^−^^56^), chronic pain (OR = 1.25; *p* = 2.32 x 10^−^^28^), alcohol-related disorders (OR = 1.35; *p* = 1.04 x 10^−^^23^), mood (OR = 1.13; *p* = 1.27 x 10^−^^22^) and anxiety (OR = 1.14; *p* = 1.00 × 10^−^^21^) disorders, viral hepatitis C (OR = 1.33; *p* = 3.04 x 10^−^^20^), and suicidal ideation or attempt (OR = 1.49; *p* = 2.17 x 10^−^^19^), amongst others (Supplementary Fig 9**;** Supplementary Table 9).

Similar patterns of association were found for the OUD-MTAG PheWAS. The top associations were with tobacco use disorder (OR = 1.30; *p* = 1.37 x 10^−^^68^), substance addiction and disorders (OR = 1.42; *p* = 1.15 x 10^−^^46^), and alcohol-related disorders (OR = 1.42; *p* = 7.36 × 10^−^^31^). OUD-MTAG also demonstrated significant associations with mood (OR = 1.12; *p* = 7.76 x 10^−^^21^) and anxiety (OR = 1.13; *p* = 1.45 x 10^−^^20^) disorders, chronic pain (OR = 1.20; *p* = 2.51 x 10^−^^20^), viral hepatitis C (OR = 1.32; *p* = 1.73 x 10^−^^18^), and suicidal ideation or attempt (OR = 1.47; *p* = 4.52 x 10^−^^18^) (Supplementary Fig 9**;** Supplementary Table 10).

### OUD polygenic risk score (PRS) analysis

In Yale-Penn 3 (YP3) EUR (*N* = 1959, 440 OUD cases), the EUR OUD PRS was a significant predictor of OUD (beta = 0.45; s.e. = 0.058; *p* = 2.9 x 10^−^^13^) with the PRS specifically accounting for 2.41% of OUD variance (Supplementary Table 11). The OUD-MTAG PRS was a stronger predictor of OUD (beta = 0.61;s.e. = 0.066; *p* = 2.0 x 10^−^^16^) explaining 3.81% of OUD variance.

In YP3 AFR (*N* = 1017, 171 OUD cases), both AFR-derived and EUR-derived OUD PRS were generated. The EUR-derived PRS (beta = 0.19;s.e. = 0.0945; *p* = 0.042; R^2^ = 0.29%) explained a small proportion of OUD variance in YP3 AFR but the AFR-derived PRS (beta = 0.073;s.e. = 0.049;*p* = 0.136; R^2^ = 0.11%) was not a significant predictor.

## Discussion

We present a large genetic study of OUD, with an overall sample size of 639,063 (EUR = 554,186; AFR = 84,877) individuals (*N*_cases_ = 20,686 [EUR = 15,251; AFR = 5435]). This study is the first to provide evidence of a GWS single-variant GWAS association between *FURIN* and OUD. We support findings from previous OUD GWAS implicating *OPRM1* as a risk locus for OUD [7], including the coding variant rs1799971 and additional *OPRM1* associations that remained statistically significant in a cross-ancestry analysis of AFR and EUR populations. We add evidence of gene-based associations with OUD and provide estimates of OUD SNP-heritability and genetic correlations with numerous traits. Further, we apply a multi-trait approach for OUD genetic discovery utilizing the high degree of genetic correlation across SUDs (OUD, AUD, CanUD) to increase power, yielding an equivalent sample size of 128,748 and 18 GWS OUD-MTAG risk loci. PheWAS of OUD and OUD-MTAG demonstrated similar patterns of associations across the phenome, and the OUD-MTAG PRS explained a larger amount of variance in OUD case status (3.81%) compared to the OUD PRS (2.41%), suggesting that the OUD-MTAG and OUD analyses are capturing similar phenomenon.

Compared to other complex psychiatric traits, there are comparatively small samples available for genetic analyses of SUDs, particularly those involving illegal substances (heroin, cocaine) [8, 9]. A strategy that increases statistical power by incorporating other sets of samples—for example, from GWAS of closely-related but non-identical traits such as other SUDs—could help advance our understanding of the genetic architecture of OUD. This study brought much more information to bear on the analysis of OUD risk variation, resulting in the identification of many more loci. These associations included two loci from the EUR OUD GWAS (*OPRM1* and *FURIN*), and 18 loci identified in the OUD-MTAG analysis (also including *FURIN*). The OUD-MTAG loci did not include *OPRM1*. The absence from the MTAG analysis of any statistically significant association mapped to *OPRM1*, a locus that should be highly-specific to OUD, was unexpected.

*FURIN* was associated with OUD risk in both SNP-based and gene-based analyses. *FURIN* (*Furin, Paired Basic Amino Acid Cleaving Enzyme*) encodes the endoprotease furin enzyme that serves a primary role in regulating synaptic neuronal activity, including the synthesis of brain-derived neurotropic factor and regulation of neurotrophin levels in the brain [26]. Genetic variation in *FURIN* has been associated with multiple psychiatric outcomes including schizophrenia [27, 28] and studies examining genetic and phenotypic features shared between schizophrenia and bipolar disorder [29, 30]. The two top *FURIN* SNPs associated with OUD are in strong LD (r^2^ = 0.91); the second strongest *FURIN* association, rs17514846, has been significantly associated with multiple cardiovascular and hypertension outcomes [31, 32], and was also GWS in a GWAS of parents’ attained age (current age of parents or parental age at death) [33]. A statistically-significant *FURIN* gene-level association being driven by rs17514846 was reported between *FURIN* and opioid addiction [34]. In a targeted follow-up in the *FURIN* gene region, there was significant association between rs11372849 (also lead SNP in the current study) and opioid addiction. Accumulating evidence linking *FURIN* and opioid outcomes, including the *FURIN* GWAS associations reported herein along with evidence of gene-based associations with opioid addiction [34], reflect the high degree of co-morbidity between OUD and psychiatric and physical health traits.

Our findings support previous OUD GWAS implicating *OPRM1* genetic variation in opioid addiction and OUD [7, 34] and extend GWS findings for *OPRM1* as a risk factor in a cross-ancestry analysis. The top association in the EUR OUD GWAS was with the *OPRM1* coding variant (rs1799971) identified in an earlier OUD GWAS, all cases of which are also included in the current study, plus an additional *OPRM1* variant (rs79704991) in low-LD with rs1799971 (r^2^ = 0.02). Two *OPRM1* variants were also GWS in the cross-ancestry OUD GWAS (rs1799971 and rs9478500; r^2^ = 0.02). Rs9478500 was previously GWS for opioid addiction in EUR [34]. There is evidence of *OPRM1*’s complex haplotype structure and the potential for multiple independent *OPRM1* risk loci influencing risk for OUD and SUDs dating to 2006 [35]. Conditional analysis of the top *OPRM1* variants (rs1799971 and rs79704991; r^2^ = 0.02) demonstrated that these variants are not independent as indicated by each variant falling below GWS when conditioned on the other; however, the variant effects remained nominally significant and there were no significant differences in the conditioned vs. unconditioned effect sizes. Future studies of larger cohorts with diverse ancestral backgrounds will be needed to distinguish the effects of OUD risk alleles across the *OPRM1* region, including the known-functional rs1799971 variant which may or may not be the variant motivating previous findings.

We found an estimated SNP-heritability (h^2^SNP) of 12.75% (Z = 11.28) compared to the previous largest OUD GWAS (h^2^SNP = 11.30%; Z = 6.27) [7]. However, the comparison between these two studies is not direct: the largest previous GWAS [7] used opioid-exposed controls, while we used a broader control definition, including not only individuals who were opioid-exposed, but subjects with no OUD assessment. This was necessitated by the fact that many of the available datasets did not define exposed controls and would have been excluded had we used the exposed control definition. Findings from the current study do not establish whether the control definition impacted the detection of genetic loci or the genetic architecture of OUD.

OUD was positively genetically correlated with other SUDs (CanUD, AUD) and psychiatric conditions (PTSD, depression, schizophrenia), with lower correlations for measures of substance use (as opposed to dependence; e.g., AUDIT-C), suggesting that OUD is more akin to measures of substance dependence than use per se. OUD was genetically correlated with multiple forms of chronic pain (e.g., lower back pain) and indicators of impairment (inability to work, decreased physical activity) and significantly genetically correlated with socioeconomic hardship (Townsend Deprivation Index) and lower levels of educational attainment. These patterns of genetic correlation are consistent with and may reflect high rates of co-occurrence of OUD with SUDs and psychiatric disorders in epidemiologic studies [25, 36]. Beyond epidemiologic estimates, SUDs and psychiatric disorders have also been demonstrated to be risk markers for severe opioid-related outcomes, such as opioid overdose [37]. Lower educational attainment and greater economic hardship have also been associated with higher rates of opioid overdose and opioid overdose-related deaths [38]. These patterns of genetic correlation are consistent with the complex clinical presentation of OUD.

We examined the utility of using MTAG to increase the information available from the limited number of genotyped OUD subjects. The OUD multi-trait analysis was feasible given the high genetic correlations with CanUD (r_g_ = 0.82) and AUD (r_g_ = 0.77) and increased by an order-of-magnitude the number of GWS risk loci detected. While this provides proof-of-concept for this approach given that many of the loci identified via OUD-MTAG have previously been implicated with psychiatric and substance use outcomes, *OPRM1* was not GWS in the OUD-MTAG analysis, so increased detection may have come at the cost of specificity for OUD. However, only 4 of the 18 OUD-MTAG GWS associations were GWS in the respective AUD and CanUD GWAS used as MTAG instruments, so the MTAG results did not simply reflect the findings from AUD and CanUD GWAS. The OUD-MTAG PRS also outperformed the OUD PRS in predicting OUD case status in the Yale-Penn holdout analysis (OUD-MTAG PRS: 3.81%, OUD PRS: 2.41%) indicating that the OUD-MTAG is capturing OUD risk, and that the OUD-MTAG PRS is more powerful than the OUD PRS alone as also reflected by the comparative number of GWS loci identified in the respective GWAS.

A PheWAS across 1291 clinical outcomes also demonstrated convergent patterns of association between OUD and OUD-MTAG with common comorbidities (including SUDs, psychiatric traits, chronic pain, viral hepatitis C), supporting that these two analyses capture genetic factors that underlie similar clinical presentations and related impairment. Additionally, summary data from the OUD MTAG analysis including multiple SUDs was highly genetically correlated (r_g_ = 0.98) with OUD [7], so it appears that the OUD-MTAG did capture genetic information relevant to OUD risk, though measuring the risk for OUD through a genetic liability for SUDs more broadly. That is, genetic risk for OUD may be a combination of a broader addiction liability (OUD-MTAG loci) combined with the opioid-specific genetic effects (e.g., *OPRM1*) that were found in the OUD single-trait analysis that are also influencing risk.

The distinction between substance-specific genetic effects and general SUD liability is of interest. Quantitative genetic studies have demonstrated both substance-specific influences, as well as heritable factors that contribute to SUDs more broadly [39, 40]. Up to 38% of variation in opioid dependence was reportedly accounted for by opioid-specific factors that were not shared with other SUDs [41]. Molecular genetic studies have begun to disentangle common vs. substance-specific genetic influences, reporting evidence to suggest the presence of a common unitary addiction factor that can account for risk across SUDs, in addition to substance-specific influences [42, 43]. Larger-scale OUD studies will be needed to parse genomic influences specific to OUD from those underlying risk for SUDs more broadly, but this will require many more genotyped OUD cases, because it cannot be accomplished via statistical methods alone.

The present study has limitations. Despite including all genotyped OUD subjects available, the OUD-only component of the present study is smaller than GWAS for other substance use behaviors [14] because OUD cases are underrepresented in available datasets. MTAG yielded a much larger sample, but at the apparent cost of a reduction in specificity marked by the non-significance of *OPRM1* in the OUD-MTAG. To maximize sample size while maintaining OUD diagnosis to define case status in extant datasets, we used an unscreened control group, which although not optimal, allowed for the inclusion of additional subject cohorts. Important consideration must be given to OUD control definitions [6, 34]. Additionally, inadequate subject numbers limited our ability to identify risk variants in non-EUR populations. This must be addressed by purposeful recruitment of AFR and other non-EUR OUD subjects.

We report novel findings from a large-scale GWAS meta-analysis of OUD and employed multi-trait approaches that advanced discovery. These identified genomic risk factors for the development of OUD and its underlying biology highlight the need to assemble large OUD datasets that include individuals from diverse ancestral backgrounds. To advance our scientific understanding of OUD risk will require study of a range of opioid-related traits (e.g., clinically diagnosed OUD, non-dependent opioid use, and prescription painkiller use) [44].

## Supplementary information

Supplemental Materials

Supplemental Figures

Supplemental Tables
