# Supplementary material for: Genome-wide association study in individuals of European and African ancestry and multi-trait analysis of opioid use disorder identifies 19 independent genome-wide significant risk loci
Source: Mol Psychiatry. 2022 Jul 25;27(10):3970–9. doi: 10.1038/s41380-022-01709-1 (PMC9718667; doi:10.1038/s41380-022-01709-1)

**Supplemental Materials**

*Phenotyping*

All cohorts used in the present analysis were assessed for a lifetime diagnosis of OUD case status. OUD case status was determined via electronic health records (EHRs) or semi-structured interviews that assessed for Diagnostic and Statistical Manual of Mental Disorders, Fourth Edition (DSM-IV) OUD criteria (**Table1**)(1). The specific EHR-based International Classification of Disease (ICD) codes and DSM-based OUD criterion used for diagnosis are described for each cohort in **Supplemental Table1**. Methods to define control status varied **(Table1).** Controls were predominately unscreened individuals with no known OUD diagnosis; from the PGC-SUD GWAS(2) we selected the comparison with the opioid-unexposed control group.

*Previously unpublished GWAS cohorts*

Previously unpublished genome-wide association studies (GWAS) of opioid use disorder (OUD) from BioVU (3), iPSYCH (4), and Yale-Penn(5,6) (Yale-Penn 3; results from Yale-Penn 1 and 2 were published previously) were included in the present OUD meta-analysis. Study descriptions are provided below.

**BioVU OUD GWAS**

 We used de-identified clinical data from Vanderbilt University Medical Center’s biobank, BioVU. We included 4,665 individuals (933 OUD cases and 3,732 controls). Controls were matched on a 1:4 ratio based on sex, race, ethnicity, median age of the longitudinal EHR measurements, and density of records (number of ICD codes and length of record). OUD status was defined as having at least one OUD ICD code. OUD controls were defined as anyone without an OUD ICD code. Details on the quality control process have been described elsewhere(7).

Genotype data were generated using the Illumina Multi-Ethnic Genotype Array (MEGAEX) for 94,474 individuals. Genotypes were filtered for SNP(<0.95) and individual (<0.98) call rates, sex discrepancies, and excessive heterozygosity(|Fhet|>0.2)(8). The sample was then filtered for cryptic relatedness by removing one individual of each pair for which pihat>0.2. PCA using FlashPCA2 combined with CEU, YRI and CHB reference sets from 1000 Genomes Project Phase 3(9) was conducted to determine European Ancestry. We confirmed the absence of genotyping batch effects using ‘batch’ as the phenotype. We used the Michigan Imputation Server with the reference panel from the Haplotype Reference Consortium. SNPs were filtered for imputation quality (R2 > 0.3 or INFO > 0.95) and converted to hard calls. We restricted to autosomal SNPs with minor allele frequency >0.01. We removed SNPs that differed by >10% from the 1000 Genomes Project phase 3 CEU set(9) and those with a Hardy Weinberg Equilibrium *p*<10^-10^. The resulting data set contained hard-called SNP information for 9,386,383 SNPs in 72,828 individuals of European Ancestry. GWAS analyses were performed using SAIGE version 0.42.1(10) and including the top 10 genetic PCs as fixed effect covariates. The project was approved by the VUMC Institutional Review Board (IRB #160302, #172020, #190418).

**iPSYCH OUD GWAS**

**Case and control definition.** Those with any of the ICD diagnoses for opioid related disorders (F11.1–F11.9) registered in the Danish hospital registers were considered as cases. The registers were followed up until Dec 2016. The controls were individuals from the iPSYCH cohort without any of the opioid related ICD diagnoses and aged at least 25 years by the end of register follow up. Individuals with and without psychiatric disorders were included in the controls. Comorbidity with psychiatric diagnoses was accounted for by including psychiatric diagnosis status for each of the disorders as binary covariates in the GWAS.

**Exclusion of related and non-European individuals.** After identifying individuals in the register with and without opioid related disorders, we excluded those who were not successfully genotyped, who were related and who were non-Europeans. The number of individuals removed at each step is shown in **Figure1**. Analytical details on the removal of related individuals and non-Europeans can be found in Pedersen et al.(4).

**Down-sampling of controls.** Since including everyone in the iPSYCH cohort without opioid related disorders as controls would result in and extreme case control ratio, we down sampled the controls to have approximately 10 times the number of cases. In total, we included 379 cases and 5221 controls in the GWAS. The down-sampling was performed separately for each of the comorbidity groups in order to maintain a similar proportion of individuals with other psychiatric disorders between cases and controls. The sample sizes split by comorbid diagnoses are shown in **Table1**.

**GWAS.** GWAS was performed using Plink using logistic regression analysis. We analyzed only variants that were retained after QC filtering (INFO>0.80, MAF>0.01, sample and variant missing rate <1% etc.) in both the iPSYCH-2012 and iPSYCH-2015i samples. This reduced the final number of variants, which was around 5 million. We used hard call genotypes for the GWAS analysis. Both the iPSYCH-2012 and iPSYCH-2015 genotype datasets were merged into a single genotype dataset and was used for GWAS analysis. This is our usual practice for GWAS where case numbers are low. In such scenarios, running GWAS separately in iPSYCH 2012 and iPSYCH 2015i is tricky due to very sparse covariates matrix that leads to too many NAs in the logistic regression. The covariates included age, sex, psychiatric diagnoses and first 10 PCs.

**Yale-Penn 3 OUD GWAS**

The Yale-Penn sample includes 11,332 genotyped and phenotyped individuals recruited across three phases (Yale-Penn 1, Yale-Penn 2, and Yale-Penn 3) according to time of recruitment and genotyping array used. For this study, data for Yale-Penn 1 and 2 was included via the PGC-SUD summary statistics. All cohorts were ascertained via recruitment at substance use treatment centers or targeted advertisements for genetic studies of cocaine, opioid, and alcohol dependence, resulting in a sample highly enriched for problematic substance use, as well as control subjects and relatives. All participants were assessed using the Semi-Structured Assessment for Drug Dependence and Alcoholism (SSADDA)(11,12). Previous analyses of OUD including Yale-Penn 1 and 2 have been published previously in studies(5,6), including those used in the PGC-SUD discovery sample(2) included in the present study; published data on Yale-Penn 3 was previously limited to use in part for replication analysis or as a target sample for polygenic risk score analyses (e.g., 6).

Yale-Penn 3 includes 3,026 genotyped and phenotyped Americans of European (EUR; N=1,986) and African (AFR; N=1,040) ancestry passing standard quality control. Genotyping was performed at the Gelernter lab at Yale University using the Illumina Multi-ethnic Global Array containing 1,779,819 markers, followed by genotype imputation using Minimac3(13) and the Haplotype Reference Consortium reference panel(14) as implemented on the Michigan imputation server (https://imputationserver.sph.umich.edu).

For the present analysis, Yale-Penn 3 EUR (N_EUR_=1,986) and AFR (N_AFR_=1,040) participants were included. DSM-IV opioid abuse and dependence diagnoses based on SSADDA assessments were used to determine OUD case status (15,16). OUD controls were defined as anyone not meeting criteria for opioid abuse or dependence. Of the 1,986 EUR participants 22.56% met criteria for OUD (N_CASE_=448); 16.54% of AFR participants (N_CASE_=172) met criteria for OUD diagnosis. The OUD GWAS was conducted using a logistic regression model in the software package GEMMA (genome-wide efficient mixed-model association)(17) accounting for subject relatedness. Covariates included age, sex, and the first 10 genetic principal components

*Previously published GWAS cohorts*

**Million Veteran Program (MVP) OUD GWAS**

The MVP is a large-scale biobank that includes U.S. Military Veteran participants across a national network of Veterans Affairs healthcare facilities. A previous MVP GWAS of OUD cases vs. opioid-exposed controls has been reported(18) that contained the same OUD cases that were included in the present analysis; however, the MVP OUD GWAS included in the current meta-analysis has not been published previously and compares OUD cases to all controls (**Table1**). OUD cases were defined as individuals with at least 1 inpatient or 2 outpatient ICD codes for OUD. MVP genotyping and imputation has been previously reported(19). OUD GWAS were conducted using logic regression in the software package PLINK, and included covariates adjusting for age, sex, and the first 10 genetic PCs. Additional information can be found in Zhou et al., 2020(18).

**Psychiatric Genomics Consortium Substance Use Disorders (PGC-SUD) OUD GWAS**

The PGC-SUD working group completed a GWAS meta-analysis across 11 studies to investigate genetic distinctions between OUD cases, opioid-exposed controls, and opioid-unexposed controls(20). For the present meta-analysis, we used summary data from the OUD case vs. opioid-unexposed control comparison groups. (**Table1**). Quality control procedures was performed using the RICOPILI pipeline(21) across all included cohorts. Ancestry-specific (African and European ancestries) GWAS were conducted across studies according to individual study design (e.g., case-control studies performed using logistic regression, family-based studies conducted using logistic mixed models). Covariates included age, sex, and 10 genetic PCs. Following cohort-specific GWAS, a sample-sized weighted meta-analysis was performed using METAL(22). Additional information can be found in Polimanti et al., 2020(20).

**Partners Biobank OUD GWAS**

A previous GWAS of OUD was performed in the Partners Healthcare System Biobank(23)—a biomedical database including clinical information and genetic data across a network of 8 Harvard-affiliated hospitals. Partners Biobank genotyping was performed using the Multi-Ethnic Global (MEG) Illumina array (Illumina, Inc., San Diego, CA) followed by imputation to the 1000 Genomes reference panel(9) using the Michigan Imputation Server. OUD case status was determined using ICD codes (**Supplemental Table1**) from patient electronic health records. GWAS were then conducted using logistic regression in PLINK v1.9(8), covarying for age, sex, and the first five genetic PCs. For additional information please see Song et al., 2020(23).

**FinnGen OUD GWAS**

FinnGen is a national research initiative in Finland aimed toward collecting genetic and electronic health information to advance personalized healthcare and improve biomedical outcomes (<https://www.finngen.fi/en/about>). Genotyping of FinnGen participants was performed using a custom ThormoFisher Axiom array containing information for 664,510 genetic variants. Clinical endpoints (e.g., OUD) are harmonized across ICD codes connected to Finnish national registries. GWAS are then performed on included traits and participants using the software package SAIGE(24) covarying for age, sex, and 10 genetic PCs. Additional information can be found at <https://finngen.gitbook.io/documentation/methods>.

*Functional characterization of identified genetic risk loci*

Variants were mapped to the nearest gene based upon physical position and further characterized using gene-mapping approaches leveraging GTEx v8(25) and BRAINEAC(26) expression quantitative trait locus (eQTL) data and 3D chromatin interactions (Hi-C)(27) as implemented in the FUMA platform (Functional Mapping and Annotation)(28).

For eQTL-based gene-mapping, GTEx v8 gene expression data included: amygdala, anterior cingulate cortex BA24, cerebellar hemisphere, cerebellum, cortex, frontal cortex BA9, hippocampus, hypothalamus, nucleus accumbens basal ganglia, putamen basal ganglia, spinal cord cervical c-1, and substantia nigra(25). BRAINEAC(26) tissues included: cerebellar cortex, frontal cortex, hippocampus, inferior olivary nucleus, occipital cortex, putamen, substantia nigra, temporal cortex, thalamus, and intralobular white matter. For Hi-C chromatin interactions, Hi-C included data from PsychENCODE(29) EP links and PsychENCODE promoter anchored loops, and FUMA-based datasets for Hi-C in adult cortex, dorsolateral prefrontal cortex, and hippocampus. Circos plots and summaries of functional characterization for all chromosomes containing genome-wide significant loci for the respective EUR OUD and OUD-MTAG GWAS can be found in **Supplemental Figures6-7** (**Supplemental Tables12-15**).

*OUD and OUD-MTAG Gene-based analysis*

GWAS summary data from the respective meta-analyses and OUD-MTAG analysis were used to carry out gene-based analyses using MAGMA (Multi-marker Analysis of GenoMic Annotation)(30) as implemented in the FUMA platform (28). Single nucleotide polymorphisms (SNPs) were mapped to 16,113 protein-coding genes based upon physical position for the OUD gene-based tests, and 15,927 genes for OUD-MTAG . GWS for the gene-based tests was defined via Bonferroni correction as *p* ≤ 3.10x10^-06^ (0.05/16,113) for OUD and *p* ≤ 3.14x10^-06^ for OUD-MTAG.

Both *FURIN* (*p*=3.09x10^-07^) and *OPRM1* (*p*=3.59x10^-07^) were significant in EUR gene-based analysis (**Supplemental Figure4**). No genes were GWS in the smaller AFR-specific analysis. In the cross-ancestry gene-based analysis, *FURIN* (*p*=6.00x10^-08^) and *OPRM1* (*p*=1.12x10^-07^) were also significant (**Supplemental Figure5**).

The OUD-MTAG gene-based analysis resulted in the identification of 66 Bonferroni significant (*p*≤0.05/15,927=3.14x10^-6^) genes (**Supplemental Figure8; Supplemental Table7**). The top OUD-MTAG gene-based association was with *PDE4B* (*p*=3.28x10^-14^). Additional significant genes of interest include *TMX2-CTNND1* (*p*=2.93x10^-11^), *HS6ST3 (p*=6.85x10^-09^), *CADM2* (*p*=1.04x10^-08^), *FURIN* (*p*=2.30x10^-08^), *FTO* (*p*=4.07x10^-08^), *DRD2* (*p*=6.25x10^-08^), and *NICN1* (*p*=7.04x10^-08^), among others**.**

*Polygenic risk score analysis*

A leave-one-out polygenic risk score (PRS) analysis was performed holding out the Yale-Penn 3 (YP3) EUR and AFR cohorts from the respective meta-analyses, allowing for examination of PRS prediction of OUD in the YP3 cohorts. New ancestry-specific OUD meta-analyses and OUD-MTAG were performed without Yale-Penn 3.

Polygenic risk scores (PRS) were then generated from the new OUD and OUD-MTAG GWAS summary statistics using PRS-CS(31) for the YP3 EUR target sample. PRS-CS(32), an extension of PRS-CS developed to improve power for PRS prediction in diverse ancestral groups, was used for the YP3 AFR target sample. PRS-CSx(32) improves statistical power by leveraging GWAS summary statistics and LD-information from multiple ancestries (EUR, AFR) to generate both EUR and AFR derived PRS.

The YP3 AFR and EUR target samples were adjusted for relatedness by removing one related individual from each relationship pair with kinship >0.0884 (second-degree relative). Variants included in the PRS were restricted to HapMap3 variants(33). Ancestry-specific 1000 Genomes references panels (EUR, AFR) were used(9). PRS were summed across chromosomes and standardized in PLINK(8). PRS were then regressed on OUD case vs. control status in the respective YP3 cohorts, covarying for age, sex, and the first 10 genetic PCs.

**References**

1. American Psychiatric Association. Diagnostic and statistical manual of mental disorders: DSM-IV-TR. Washington, DC: American Psychiatric Association 2000.
2. Polimanti R, Walters RK, Johnson EC, Mcclintick JN, Adkins AE, Adkins DE, et al. Leveraging genome-wide data to investigate differences between opioid use vs. opioid dependence in 41,176 individuals from the Psychiatric Genomics Consortium. *Molecular Psychiatry* 2020; 25: 1673–1687.
3. Roden D, Pulley J, Basford M, Bernard G, Clayton E, Balser J, et al. Development of a Large-Scale De-Identified DNA Biobank to Enable Personalized Medicine. *Clinical Pharmacology & Therapeutics* 2008; 84: 362–369.
4. Pedersen CB, Bybjerg-Grauholm J, Pedersen MG, Grove J, Agerbo E, Bækvad-Hansen M, et al. The iPSYCH2012 case–cohort sample: new directions for unravelling genetic and environmental architectures of severe mental disorders. *Molecular Psychiatry* 2018; 23: 6–14.
5. Sherva R, Wang Q, Kranzler H, Zhao H, Koesterer R, Herman A, et al. Genome-wide Association Study of Cannabis Dependence Severity, Novel Risk Variants, and Shared Genetic Risks. *JAMA Psychiatry* 2016; 73: 472.
6. Zhou H, Rentsch CT, Cheng Z, Kember RL, Nunez YZ, Sherva RM, et al. Association of OPRM1 Functional Coding Variant With Opioid Use Disorder. *JAMA Psychiatry* 2020; 77: 1072.
7. Dennis JK, Sealock JM, Straub P, Lee YH, Hucks D, Actkins K et al. Clinical laboratory test-wide association scan of polygenic scores identifies biomarkers of complex disease. *Genome Medicine* 2021; 13. doi: 10.1186/s13073-020-00820-8.
8. Purcell S, Neale B, Todd-Brown K, Thomas L, Ferreira MAR, Bender D. PLINK: A Tool Set for Whole-Genome Association and Population-Based Linkage Analyses. *The American Journal of Human Genetics* 2007; 81: 559–575.
9. Auton A, Abecasis GR, Altshuler DM, Durbin RM, Abecasis GR, Bentley DR, et al. A global reference for human genetic variation. *Nature* 2015; 526: 68–74.
10. Zhou W, Nielsen JB, Fritsche LG, Dey R, Gabrielsen ME, Wolford BN, et al. Efficiently controlling for case-control imbalance and sample relatedness in large-scale genetic association studies. *Nature Genetics* 2018; 50: 1335–1341.
11. Pierucci-Lagha A, Gelernter J, Chan G, Arias A, Cubells JF, Farrer L, et al. Reliability of DSM-IV diagnostic criteria using the semi-structured assessment for drug dependence and alcoholism (SSADDA). *Drug and Alcohol Dependence* 2007; 91: 85–90.
12. Pierucci-Lagha A, Gelernter J, Feinn R, Cubells JF, Pearson D, Pollastri A, et al. Diagnostic reliability of the Semi-structured Assessment for Drug Dependence and Alcoholism (SSADDA). *Drug and Alcohol Dependence* 2005; 80: 303–312.
13. Das S, Forer L, Schönherr S, Sidore C, Locke AE, Kwong A, et al. Next-generation genotype imputation service and methods. *Nature Genetics* 2016; 48: 1284–1287.
14. McCarthy S, Das S, Kretzschmar W, Delaneau O, Wood AR, Teumer A, et al. A reference panel of 64,976 haplotypes for genotype imputation. *Nature Genetics* 2016; 48: 1279–1283.
15. Cheng Z, Yang BZ, Zhou H, Nunez Y, Kranzler HR, Gelernter J. Genome-wide scan identifies opioid overdose risk locus close to MCOLN1. *Addiction biology* 2020; 2019:e12811. Epub 2019/07/31.
16. Cheng Z, Zhou H, Sherva R, Farrer LA, Kranzler HR, Gelernter J. Genome-wide Association Study Identifies a Regulatory Variant of RGMA Associated With Opioid Dependence in European Americans. *Biological Psychiatry* 2018; 84: 762–770.
17. Zhou X, Stephens M. Genome-wide efficient mixed-model analysis for association studies. *Nature Genetics* 2012; 44: 821–824.
18. Zhou H, Rentsch CT, Cheng Z, Kember RL, Nunez YZ, Sherva RM, et al. Association of OPRM1 Functional Coding Variant With Opioid Use Disorder. *JAMA Psychiatry* 2020; 77: 1072.
19. Gaziano JM, Concato J, Brophy M, Fiore L, Pyarajan S, Breeling J, et al. Million Veteran Program: A mega-biobank to study genetic influences on health and disease. *Journal of Clinical Epidemiology* 2016; 70: 214–223.
20. Polimanti R, Walters RK, Johnson EC, Mcclintick JN, Adkins AE, Adkins DE, et al. Leveraging genome-wide data to investigate differences between opioid use vs. opioid dependence in 41,176 individuals from the Psychiatric Genomics Consortium. *Molecular Psychiatry* 2020; 25: 1673–1687.
21. Lam M, Awasthi S, Watson HJ, Goldstein J, Panagiotaropoulou G, Trubetskoy V, et al. RICOPILI: Rapid Imputation for COnsortias PIpeLIne. *Bioinformatics* 2020; 36: 930–933.
22. Willer CJ, Li Y, Abecasis GR. METAL: fast and efficient meta-analysis of genomewide association scans. *Bioinformatics* 2010; 26: 2190–2191.
23. Song W, Kossowsky J, Torous J, Chen C-Y, Huang H, Mukamal KJ, et al. Genome-wide association analysis of opioid use disorder: A novel approach using clinical data. *Drug and Alcohol Dependence* 2020; 217: 108276.
24. Zhou W, Nielsen JB, Fritsche LG, Dey R, Gabrielsen ME, Wolford BN, et al. Efficiently controlling for case-control imbalance and sample relatedness in large-scale genetic association studies. *Nature Genetics* 2018; 50: 1335–1341.
25. Lonsdale J, Thomas J, Salvatore M, Phillips R, Lo E, Shad S, et al. The Genotype-Tissue Expression (GTEx) project. *Nature Genetics* 2013; 45: 580–585.
26. Ramasamy A, Trabzuni D, Guelfi S, Varghese V, Smith C, Walker R, et al. Genetic variability in the regulation of gene expression in ten regions of the human brain. *Nature Neuroscience* 2014; 17: 1418–1428.
27. Schmitt A, Hu M, Jung I, Xu Z, Qiu Y, Catherine, et al. A Compendium of Chromatin Contact Maps Reveals Spatially Active Regions in the Human Genome. *Cell Reports* 2016; 17: 2042–2059.
28. Watanabe K, Taskesen E, Van Bochoven A, Posthuma D. Functional mapping and annotation of genetic associations with FUMA. *Nature Communications* 2017; 8. doi: 10.1038/s41467-017-01261-5.
29. Akbarian S, Liu C, Knowles JA, Vaccarino FM, Farnham PJ, Crawford GE, et al. The PsychENCODE project. *Nature Neuroscience* 2015; 18: 1707–1712.
30. De Leeuw CA, Mooij JM, Heskes T, Posthuma D. MAGMA: Generalized Gene-Set Analysis of GWAS Data. *PLOS Computational Biology* 2015; 11: e1004219.
31. Ge T, Chen C-Y, Ni Y, Feng Y-CA, Smoller JW. Polygenic prediction via Bayesian regression and continuous shrinkage priors. *Nature Communications* 2019; 10. doi: 10.1038/s41467-019-09718-5.
32. Ruan Y, Lin Y-F, Feng Y-CA, Chen C-Y, Lam M, Guo Z, et al. Improving polygenic prediction in ancestrally diverse populations. *Nature Genetics* 2022; doi:10.1038/s41588-022-01054-7
33. International HapMap 3 Consortium. Integrating common and rare genetic variation in diverse human populations. *Nature* 2010; 467: 52–58.

**Table Legends**

**Supplemental Materials Table1.** IPSYCH GWAS sample sizes split by comorbid diagnoses.

**Figure Legends**

**Supplemental Materials Figure1.** IPSYCH GWAS EUR sample inclusion pipeline.

**Supplemental Materials Table1. IPSYCH GWAS sample sizes split by comorbid diagnoses.**

|  | **With OUD** | **Without OUD** | **Down sampled controls** | **N in full final sample** |
| --- | --- | --- | --- | --- |
| Controls | 40 | 15580 | 400 | 400 |
| ADHD | 121 | 6131 | 1210 | 1468 |
| ASD | 17 | 3515 | 170 | 381 |
| Schizophrenia | 127 | 4023 | 1270 | 1555 |
| Bipolar | 34 | 2140 | 340 | 579 |
| MDD | 183 | 20429 | 1830 | 2889 |
| Anorexia | 15 | 2915 | 150 | 268 |

**Supplemental Materials Figure1. IPSYCH GWAS EUR sample inclusion pipeline.**


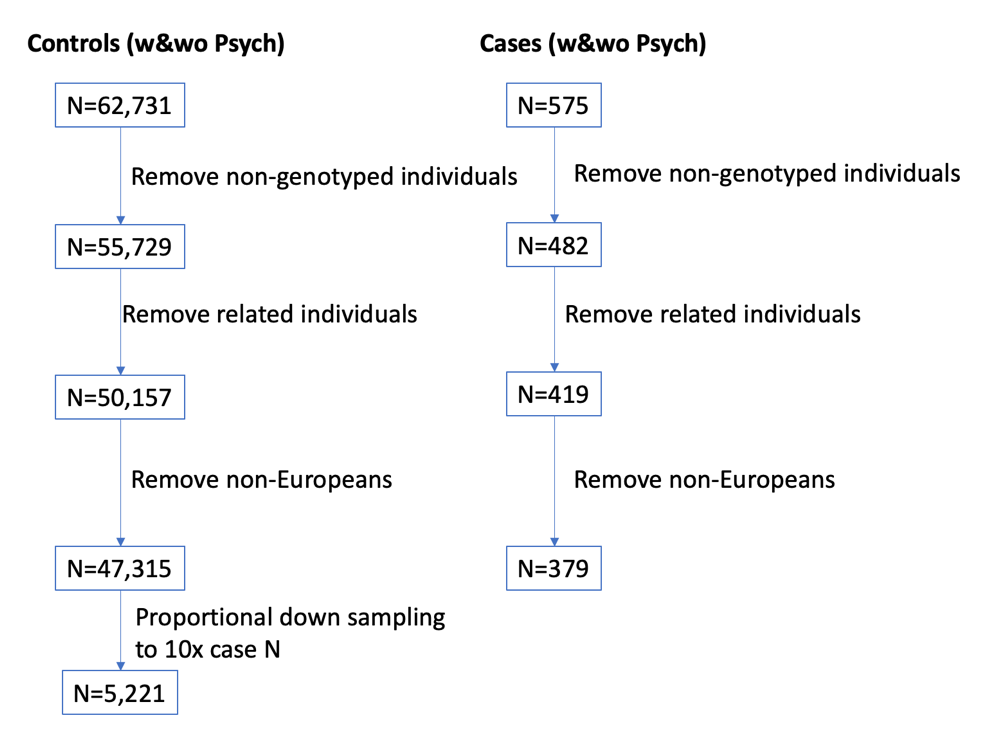

Supplement: Supplementary file 1 — Supplemental Materials [file 41380_2022_1709_MOESM1_ESM.docx]
