# Supplementary material for: Genome-wide association study in individuals of European and African ancestry and multi-trait analysis of opioid use disorder identifies 19 independent genome-wide significant risk loci
Source: Mol Psychiatry. 2022 Jul 25;27(10):3970–9. doi: 10.1038/s41380-022-01709-1 (PMC9718667; doi:10.1038/s41380-022-01709-1)
Supplement: Supplementary file 2 — Supplemental Figures [file 41380_2022_1709_MOESM2_ESM.docx]

**Supplemental Figures**

**Supplemental Figure 1.** Regional plots of *OPRM1* gene region on chromosome 6 for EUR OUD GWAS top *OPRM1* associations (a) rs1799971 (*p*=4.92x10^-09^ ) and (b) rs79704991 (*p*=1.11x10^-08^; r^2^=0.02).

**Supplemental Figure 2.** Manhattan plot of African-ancestry OUD GWAS results.

**Supplemental Figure 3.** Manhattan plot of cross-ancestry OUD GWAS results.

**Supplemental Figure 4.** Manhattan plot of EUR ancestry OUD gene-based results.

**Supplemental Figure 5.** Manhattan plot of cross-ancestry OUD gene-based results.

**Supplemental Figure 6.** Circos plots for chromosomes containing genome-wide significant loci for the OUD EUR GWAS.

**Supplemental Figure 7.** Circos plots for chromosomes containing genome-wide significant loci for the OUD-MTAG GWAS.

**Supplemental Figure 8.** Manhattan plot of OUD-MTAG gene-based results.

**Supplemental Figure 9.** BioVU PheWAS results for EUR OUD GWAS (OUD-META; left panel) and OUD multi-trait analysis (OUD-MTAG; right panel).

**Supplemental Figure 1. Regional plots of *OPRM1* gene region on chromosome 6 for EUR OUD GWAS top *OPRM1* associations (a) rs1799971 (*p*=4.92x10^-09^ ) and (b) rs79704991 (*p*=1.11x10^-08^; r^2^=0.02).**

**(a)**

**
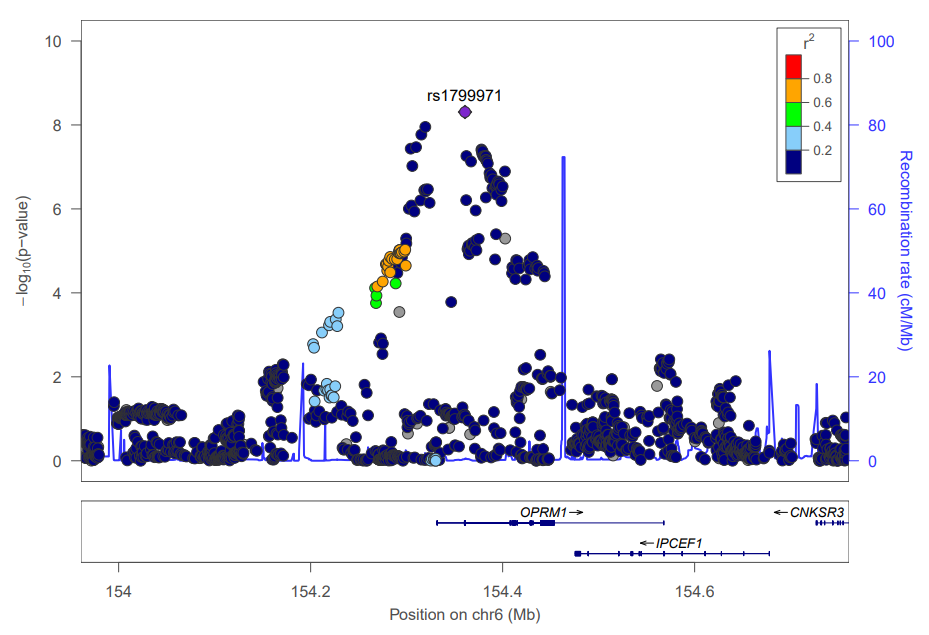
**

**(b)**

**
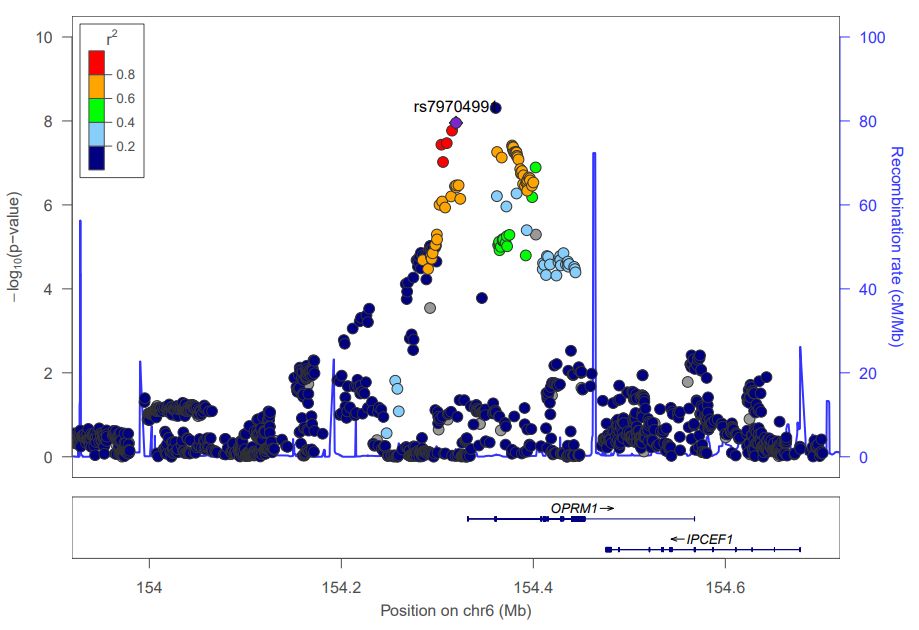
**

**Supplemental Figure 2. Manhattan plot of African-ancestry OUD GWAS results**

**
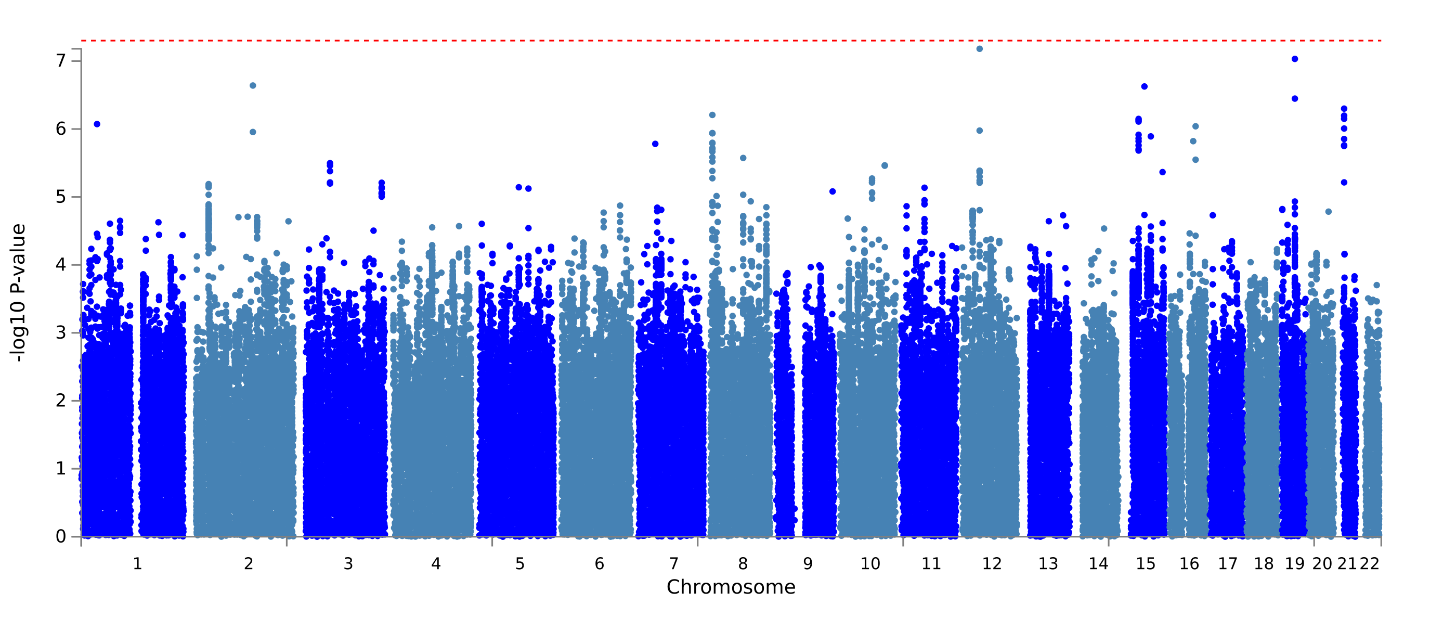
**

**Supplemental Figure 3. Manhattan plot of cross-ancestry OUD GWAS results.**

**
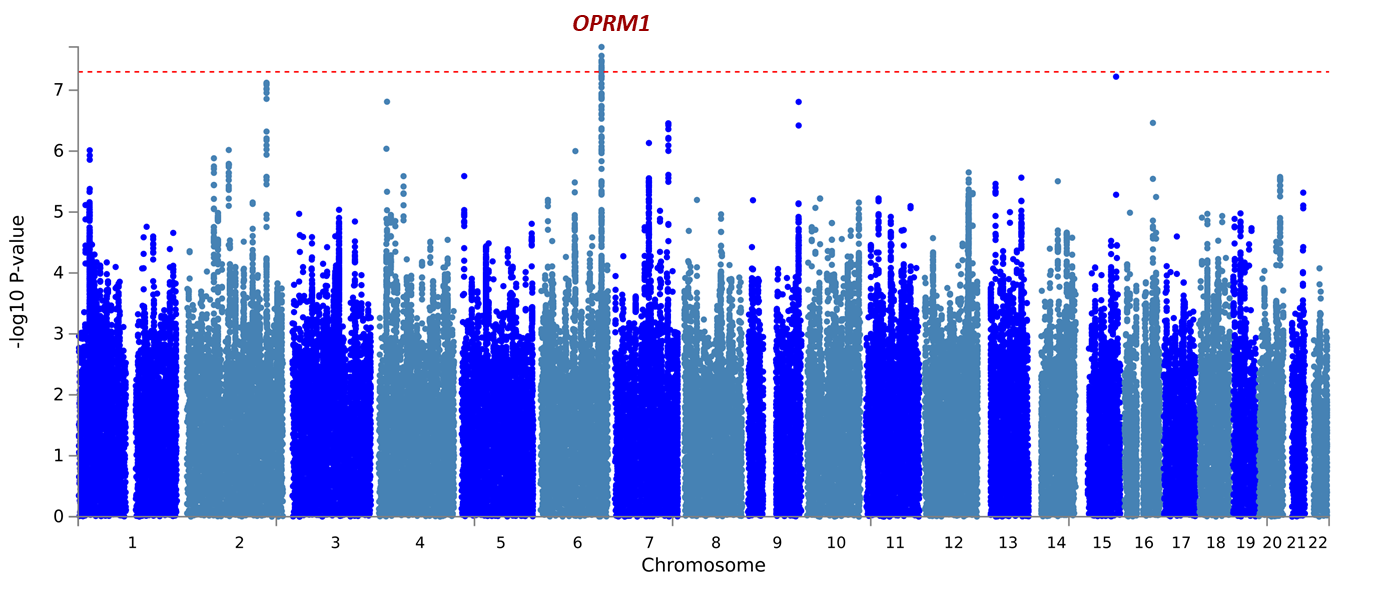
**

**Supplemental Figure 4. Manhattan plot of EUR ancestry OUD gene-based results.**

**
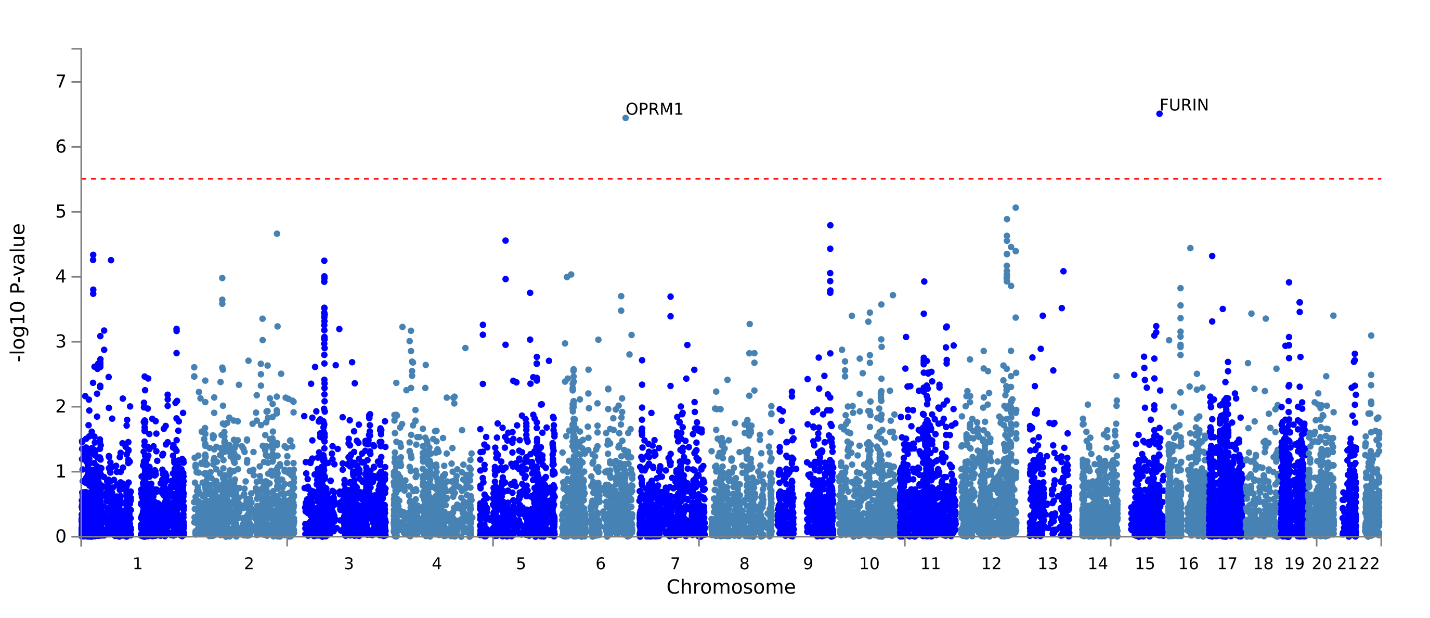
**

**Supplemental Figure 5. Manhattan plot of cross-ancestry OUD gene-based results.**

**
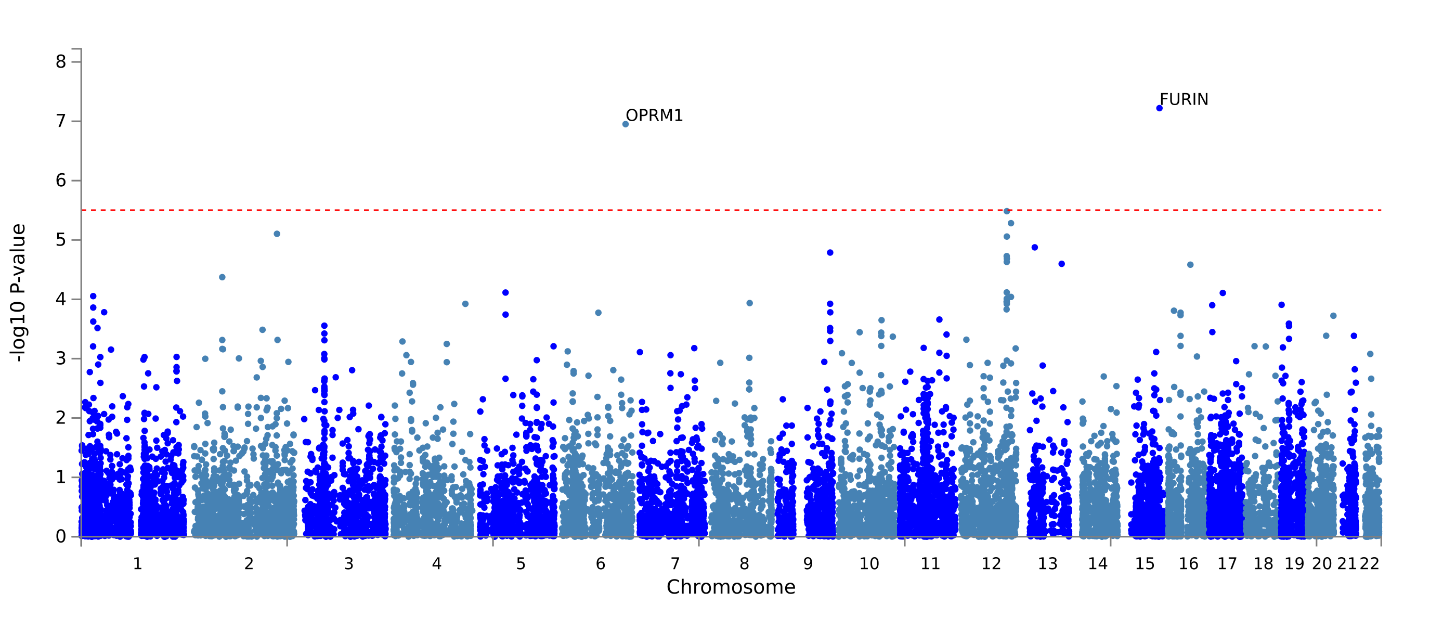
**

**Supplemental Figure 6. Circos plots for chromosomes containing genome-wide significant loci for the OUD EUR GWAS.**

**Note. Outer most layer** is a manhattan plot of genome-wide association study (GWAS) single-nucleotide polymorphisms (SNPs) with *p*≤0.05. SNPs are plotted by chromosomal position along the *x*-axis with their corresponding -log^-10^ *p*-value on the *y*-axis. Linkage-disequilibrium (LD) between the identified lead SNP and surrounding SNPs is indicated from r^2^>0.8 (red), r^2^>0.6 (orange), r^2^>0.4 (green), r^2^>0.2 (blue). SNPs that are not in LD with the lead SNP (r^2^≤0.02) are gray. **Second layer (chromosome ring):** Chromosomal regions containing identified genomic risk loci are colored in blue. The names of genes implicated based upon variant associations with brain tissue expression quantitative trait loci (eQTLs) are colored green. The names of genes implicated based upon 3D chromatin interactions (Hi-C) are colored orange. Genes that are mapped based upon both eQTLs and Hi-C associations are colored red. **Third layer (chromosome ring):** Variants mapped to genes based upon associations with brain tissue eQTLs are linked in green. Variants mapped to genes based upon Hi-C data are linked in orange.

1. **Chromosome 6**

**
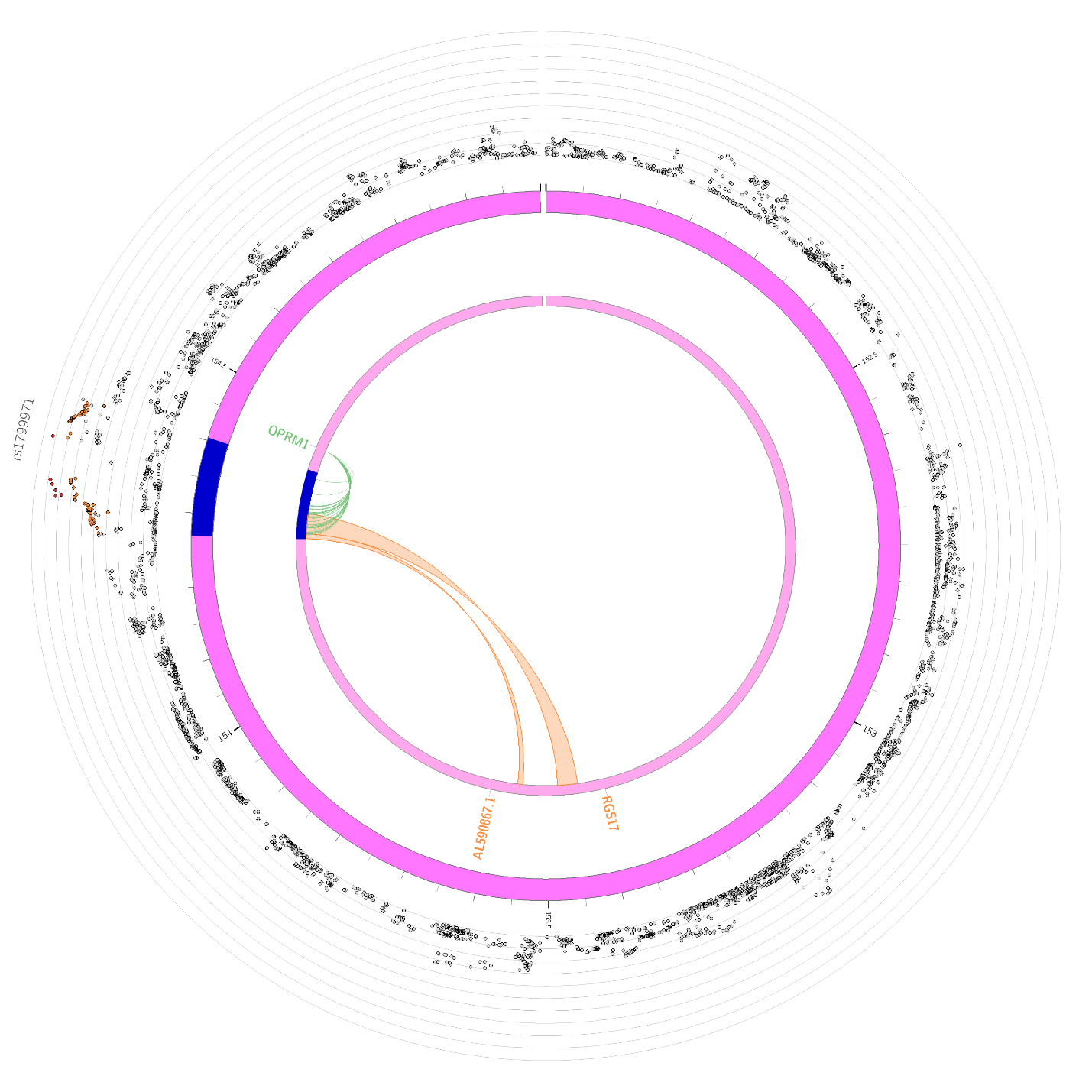
**

1. **Chromosome 15**

**
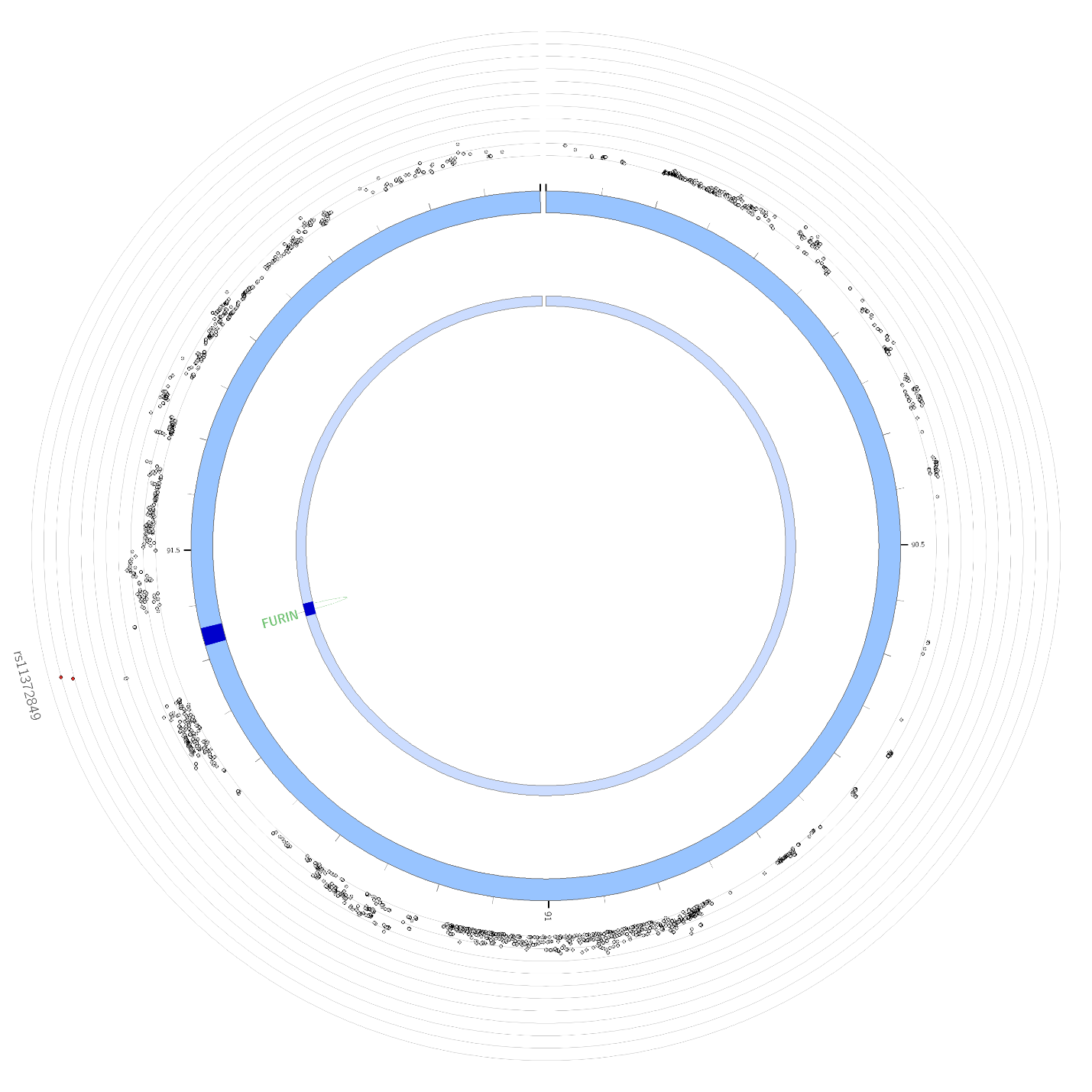
**

**Supplemental Figure 7. Circos plots for chromosomes containing genome-wide significant loci for the OUD-MTAG GWAS.**

**Note. Outer most layer** is a manhattan plot of genome-wide association study (GWAS) single-nucleotide polymorphisms (SNPs) with *p*≤0.05. SNPs are plotted by chromosomal position along the *x*-axis with their corresponding -log^-10^ *p*-value on the *y*-axis. Linkage-disequilibrium (LD) between the identified lead SNP and surrounding SNPs is indicated from r^2^>0.8 (red), r^2^>0.6 (orange), r^2^>0.4 (green), r^2^>0.2 (blue). SNPs that are not in LD with the lead SNP (r^2^≤0.02) are gray. **Second layer (chromosome ring):** Chromosomal regions containing identified genomic risk loci are colored in blue. The names of genes implicated based upon variant associations with brain tissue expression quantitative trait loci (eQTLs) are colored green. The names of genes implicated based upon 3D chromatin interactions (Hi-C) are colored orange. Genes that are mapped based upon both eQTLs and Hi-C associations are colored red. **Third layer (chromosome ring):** Variants mapped to genes based upon associations with brain tissue eQTLs are linked in green. Variants mapped to genes based upon Hi-C data are linked in orange.

1. **Chromosome 1**

**
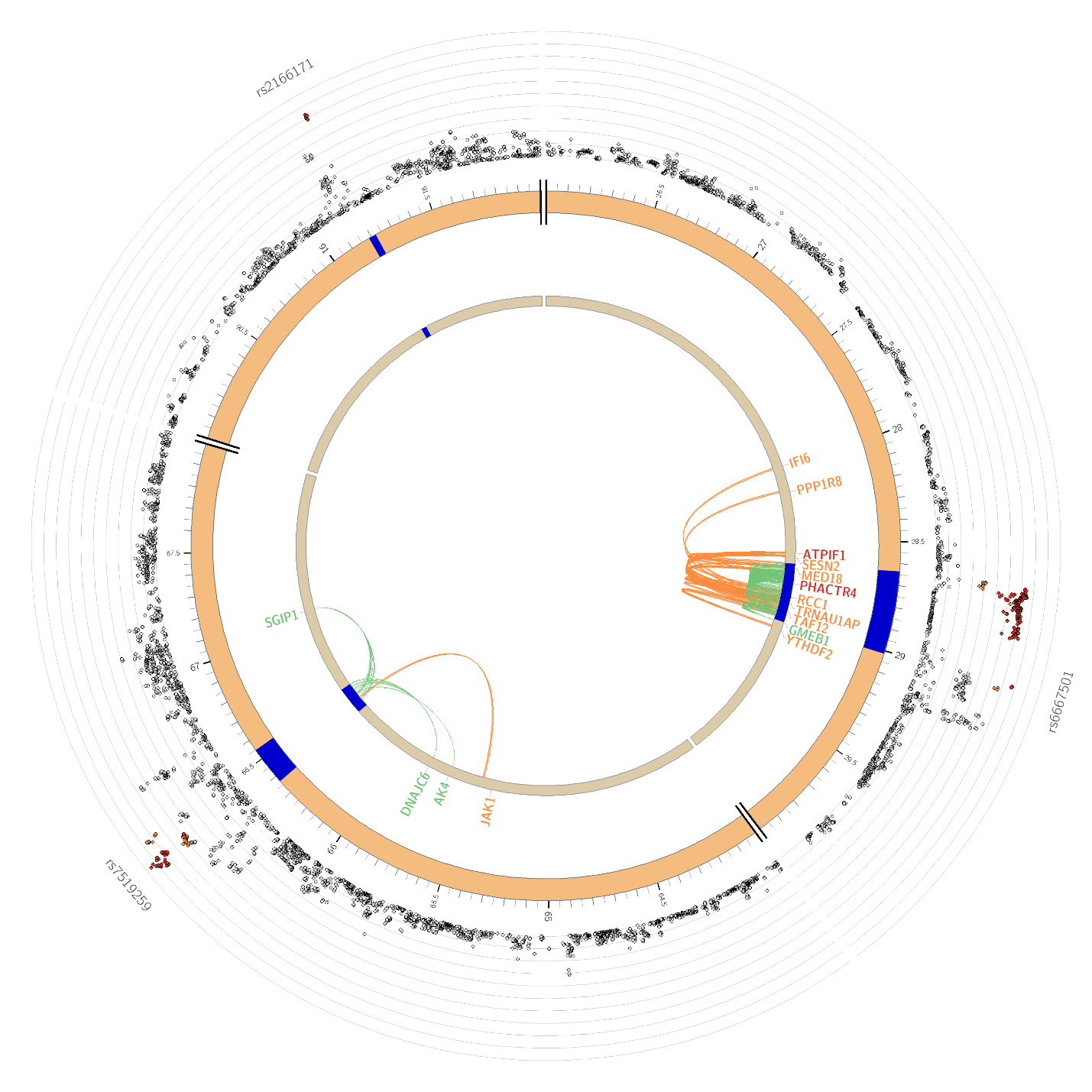
**

1. **Chromosome 3**

**
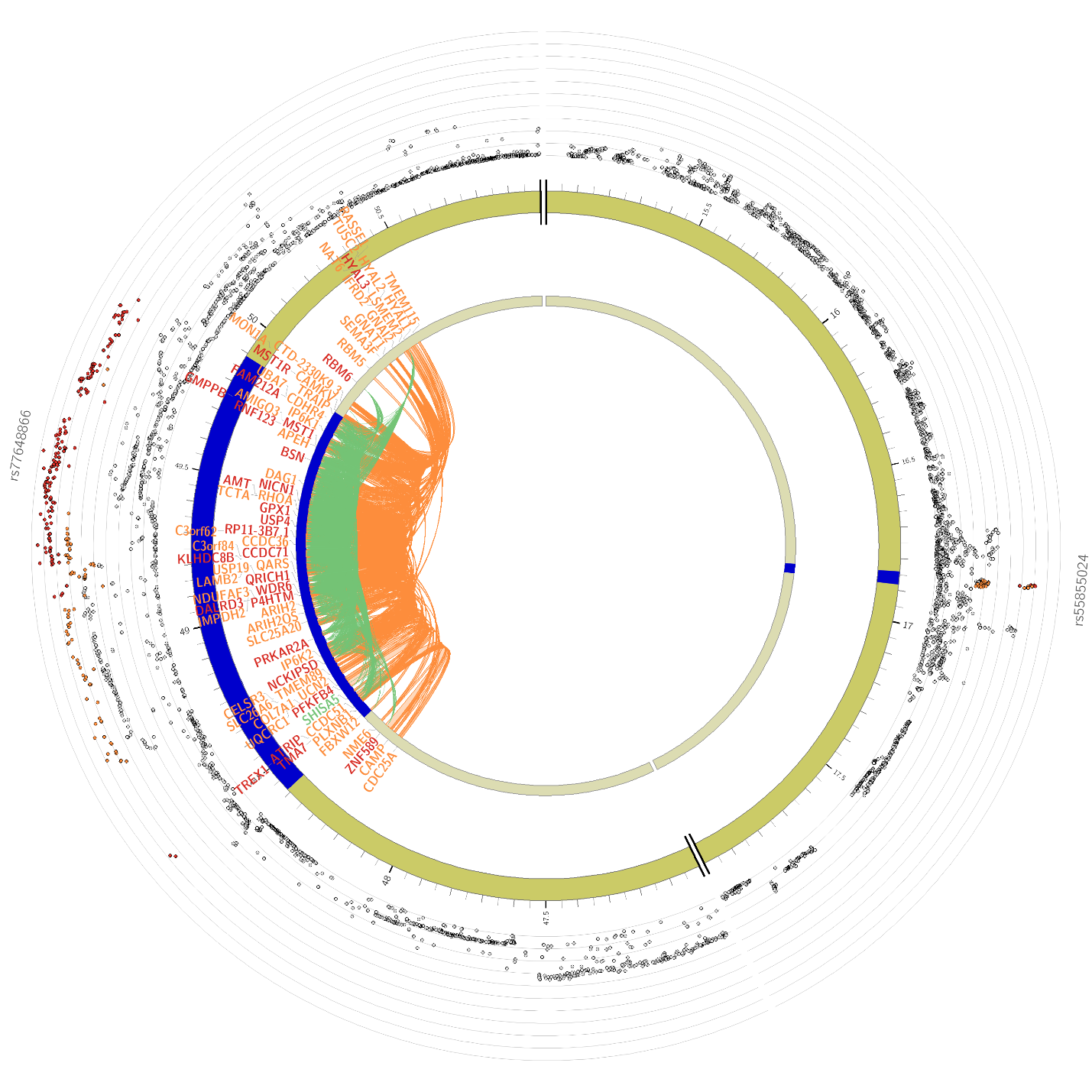
**

1. **Chromosome 4**

**
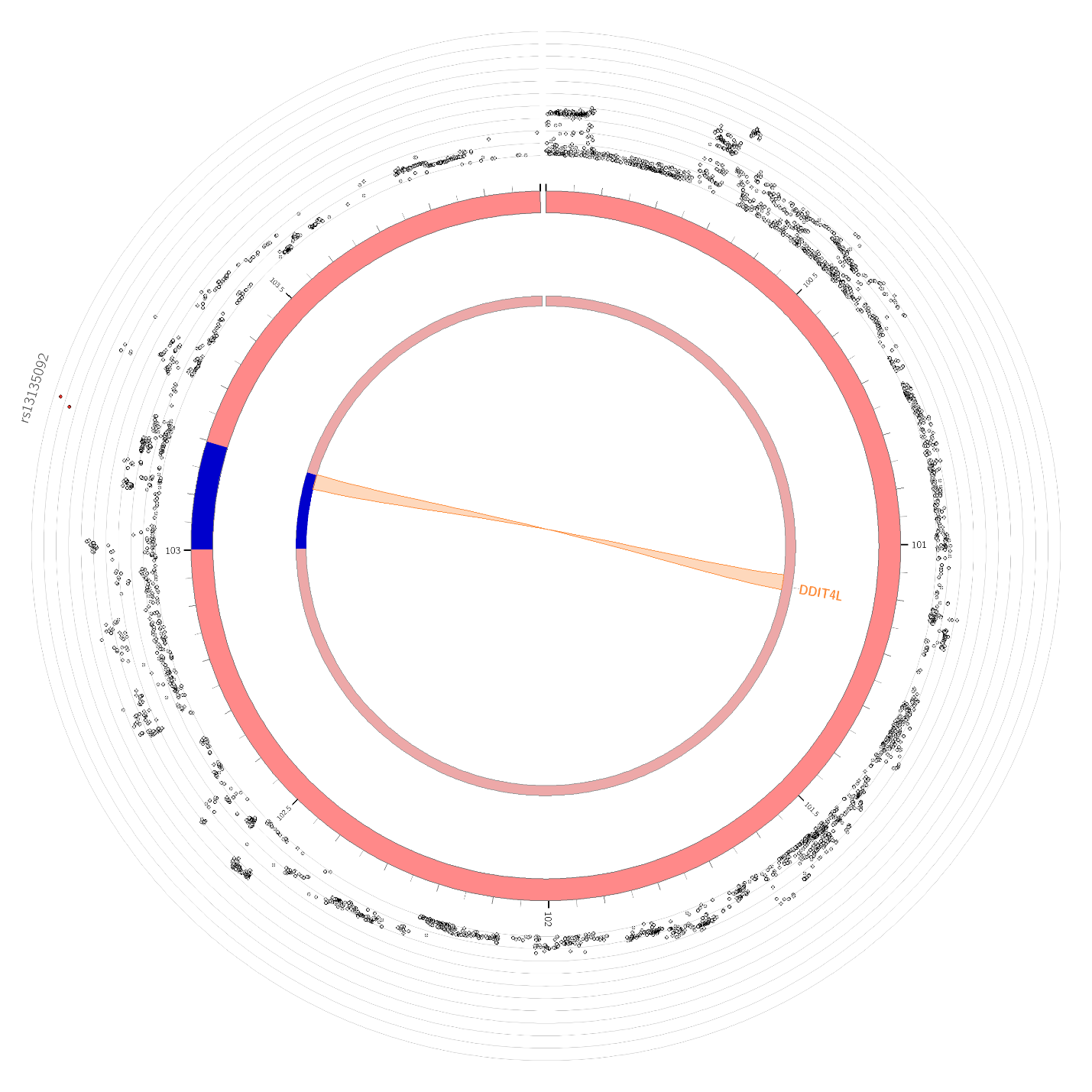
**

1. **Chromosome 6**

**
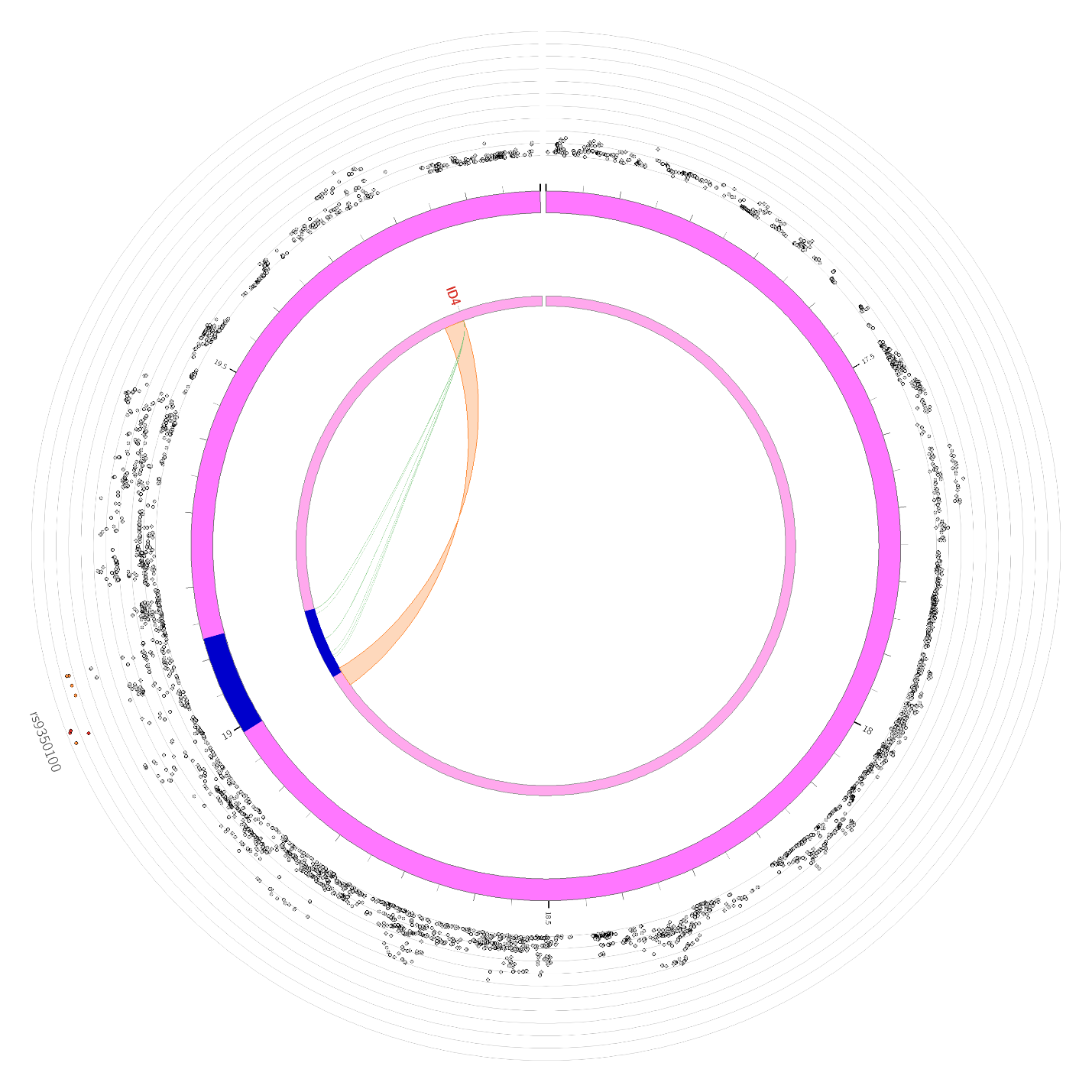
**

1. **Chromosome 7**

**
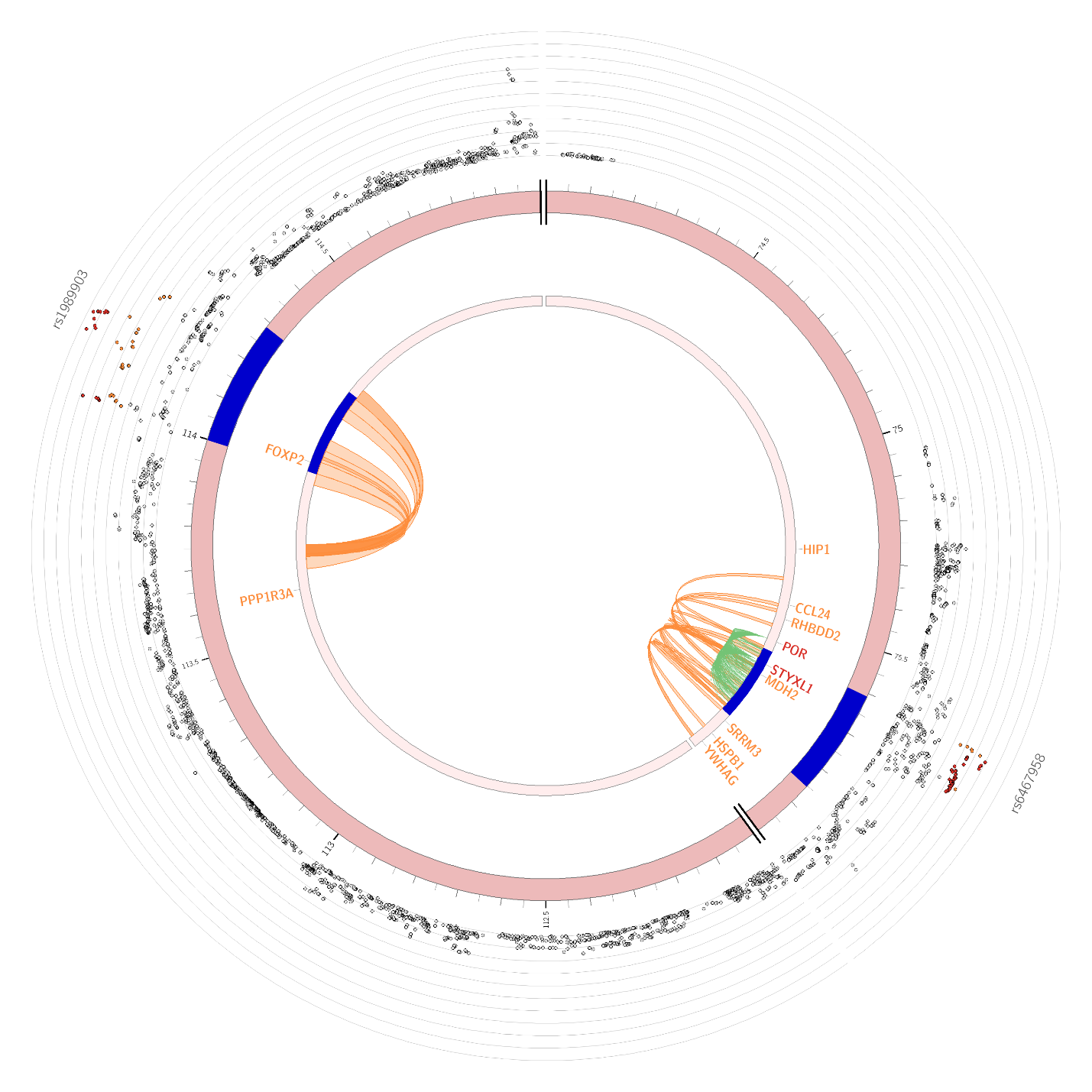
**

1. **Chromosome 9**

**
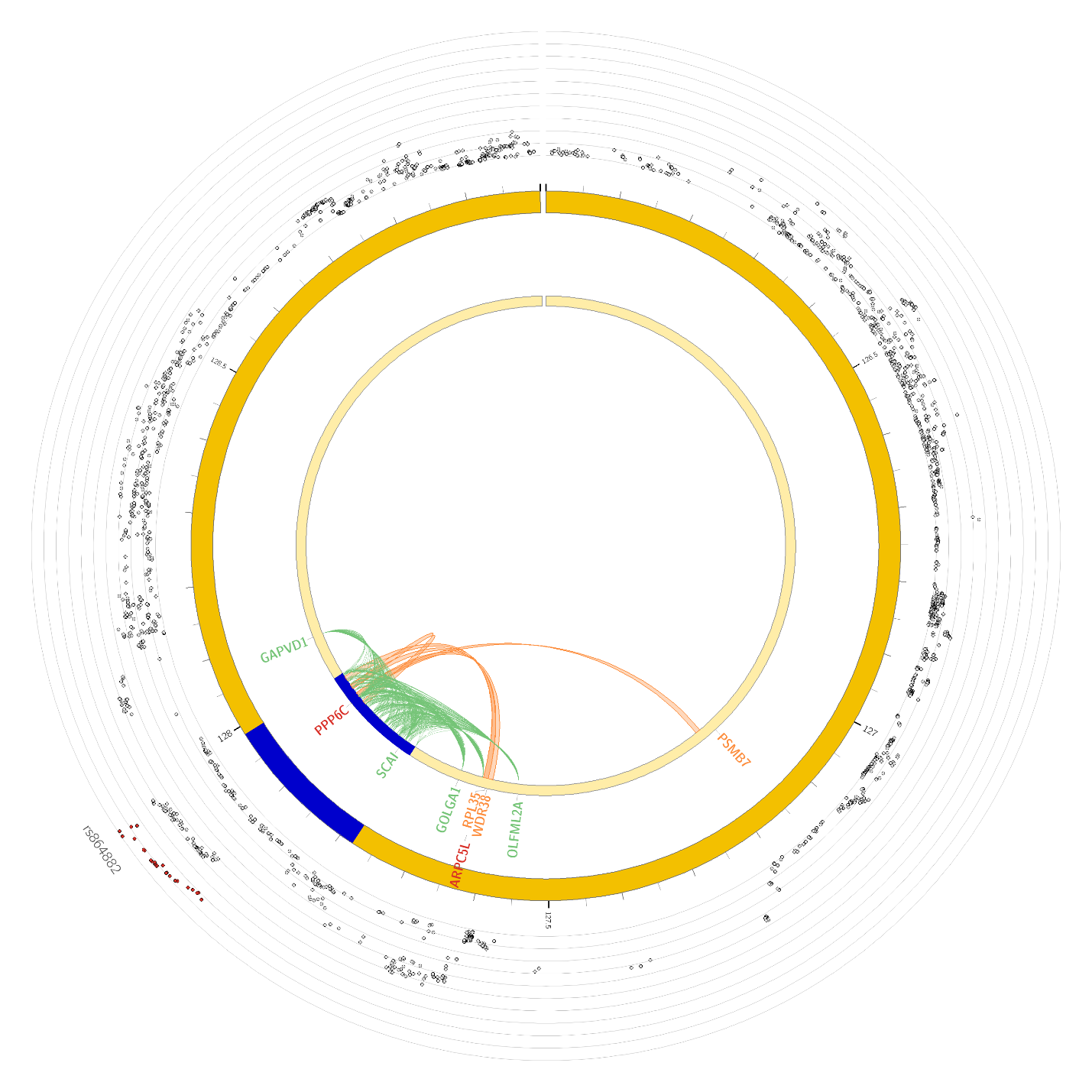
**

1. **Chromosome 11**

**
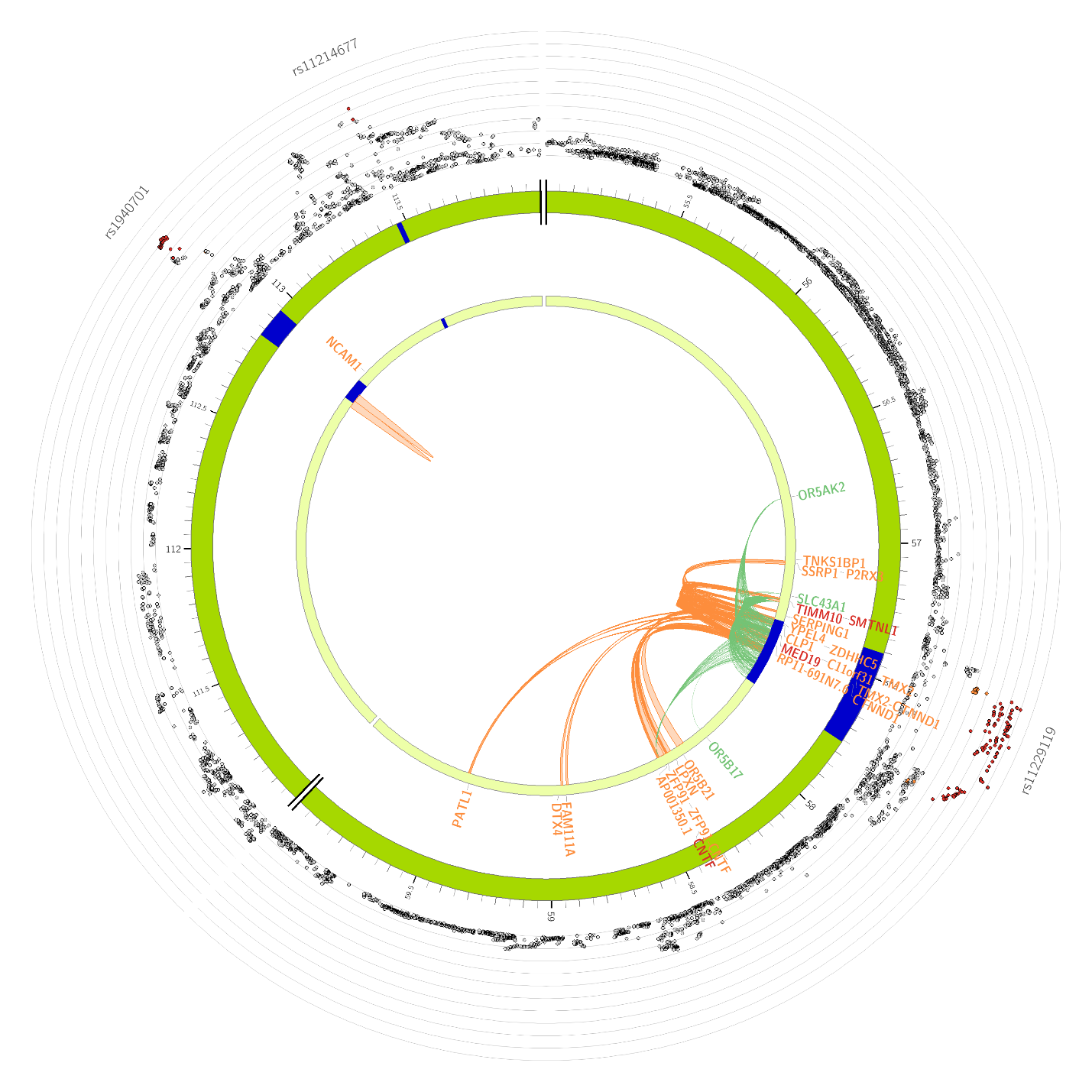
**

1. **Chromosome 13**

**
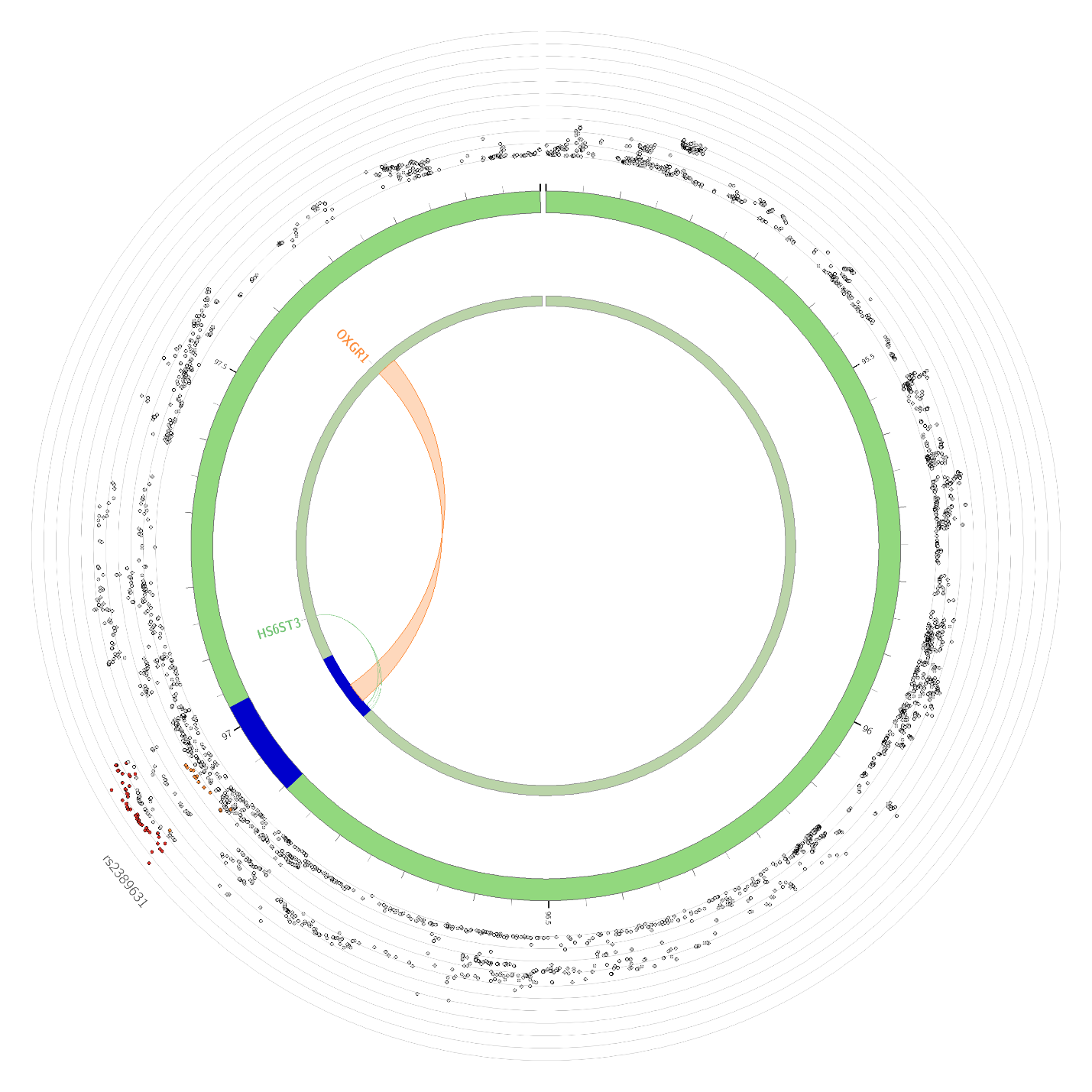
**

1. **Chromosome 15**

**
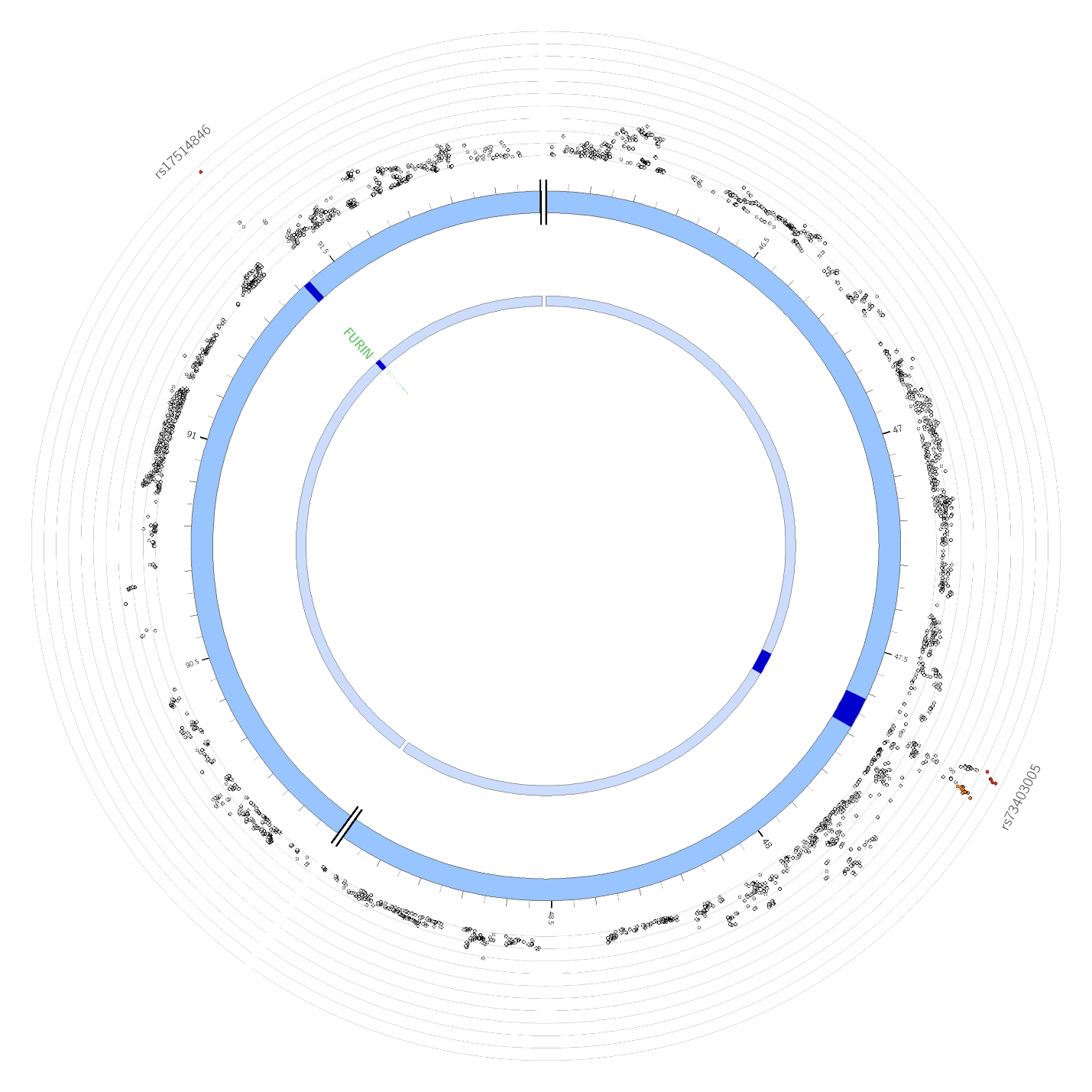
**

1. **Chromosome 16**

**
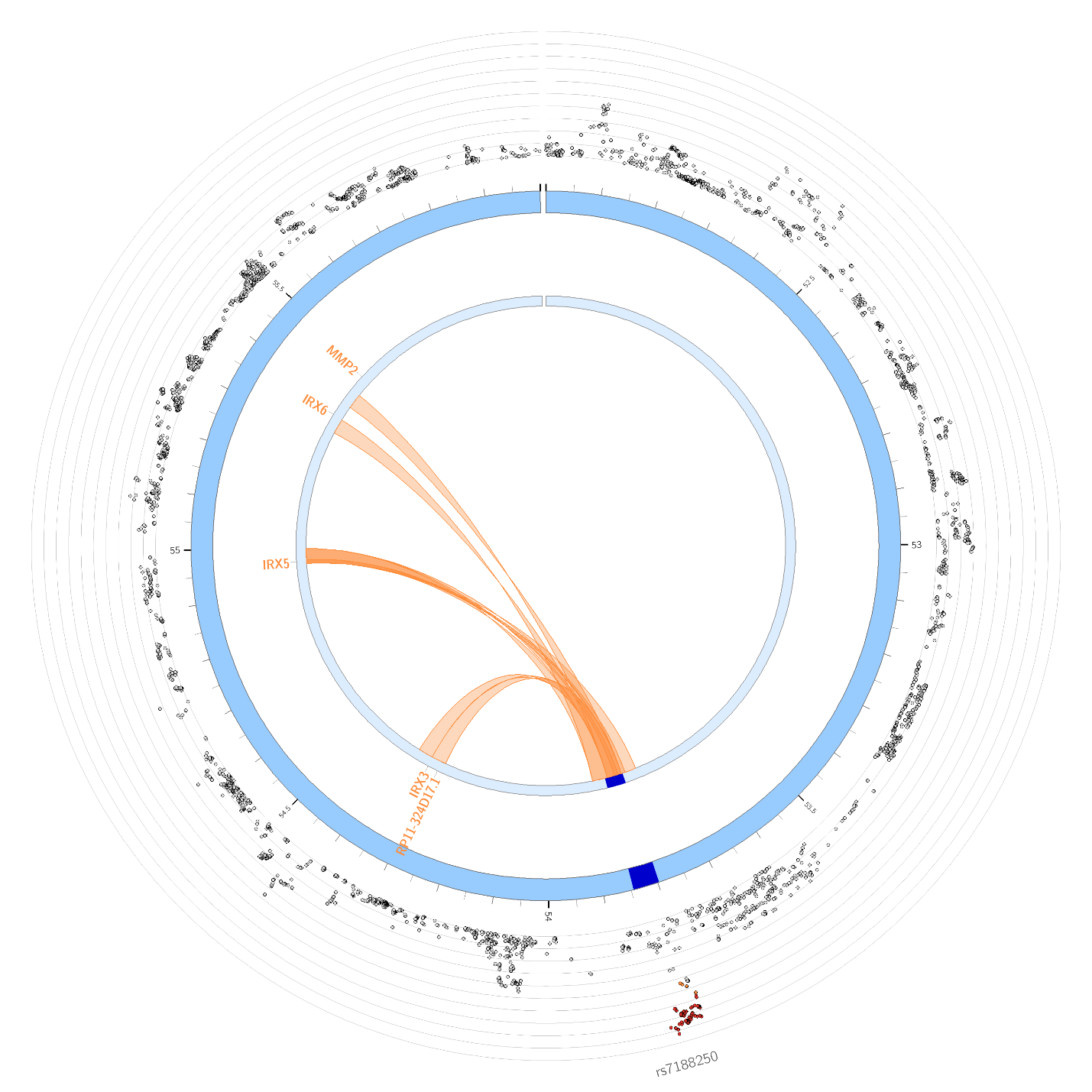
**

1. **Chromosome 19**

**
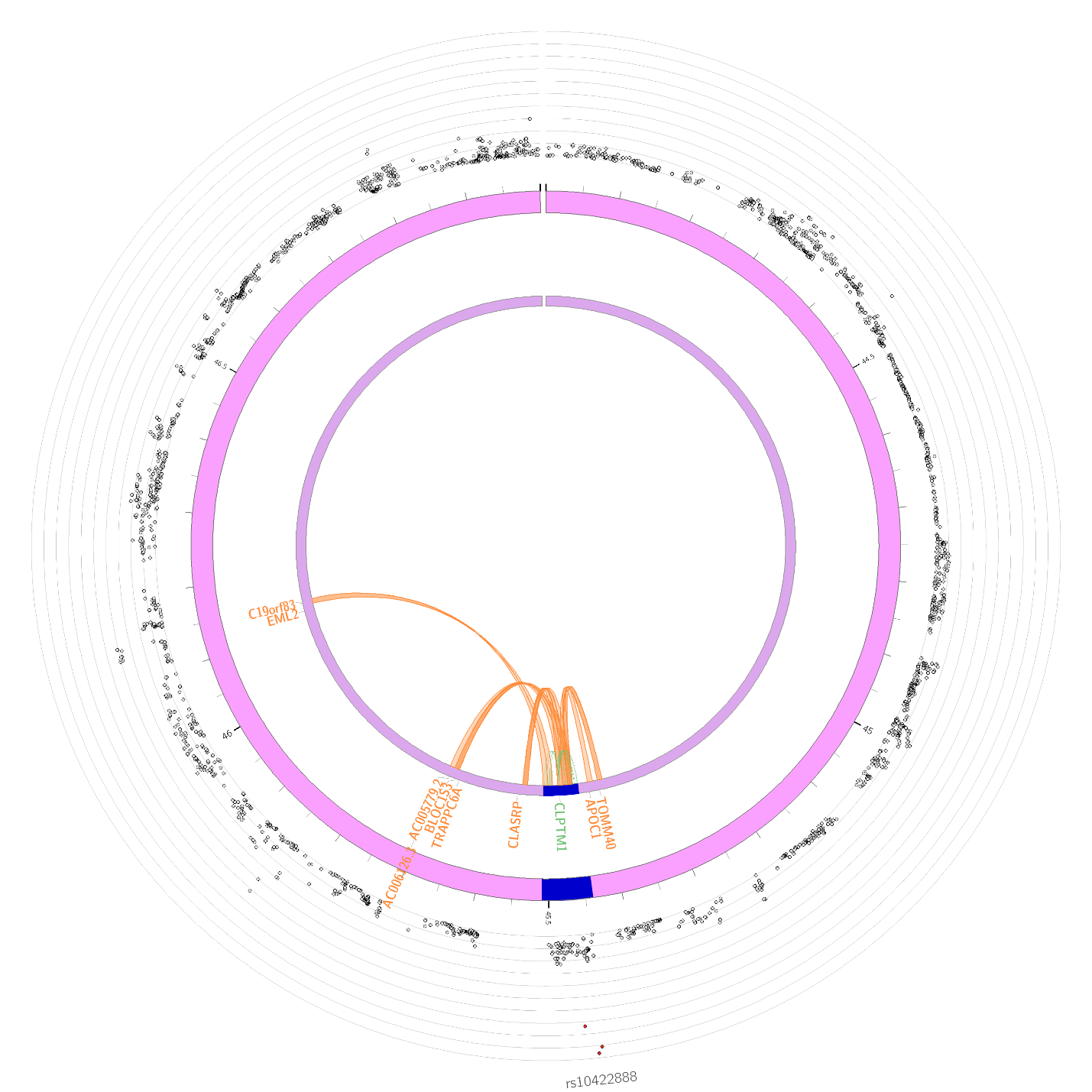
**

**Supplemental Figure 8. Manhattan plot of OUD-MTAG gene-based results.**

**
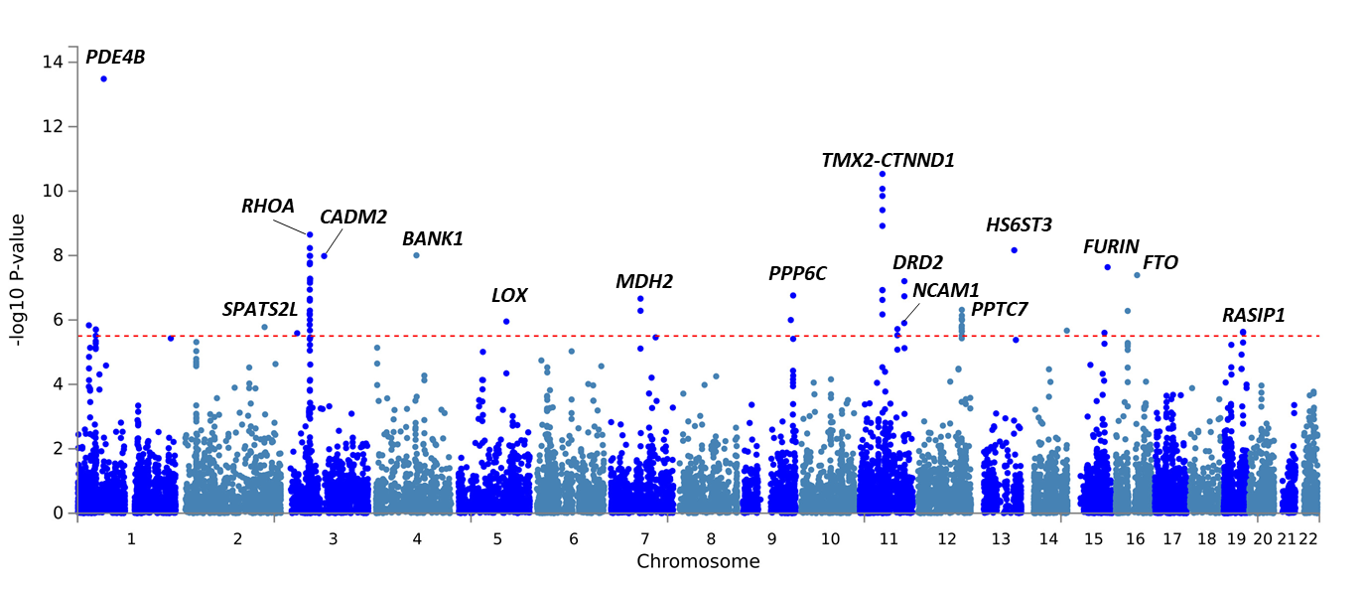
**

**Supplemental Figure 9. BioVU PheWAS results for EUR OUD GWAS (OUD-META; left panel) and OUD multi-trait analysis (OUD-MTAG; right panel).**

**
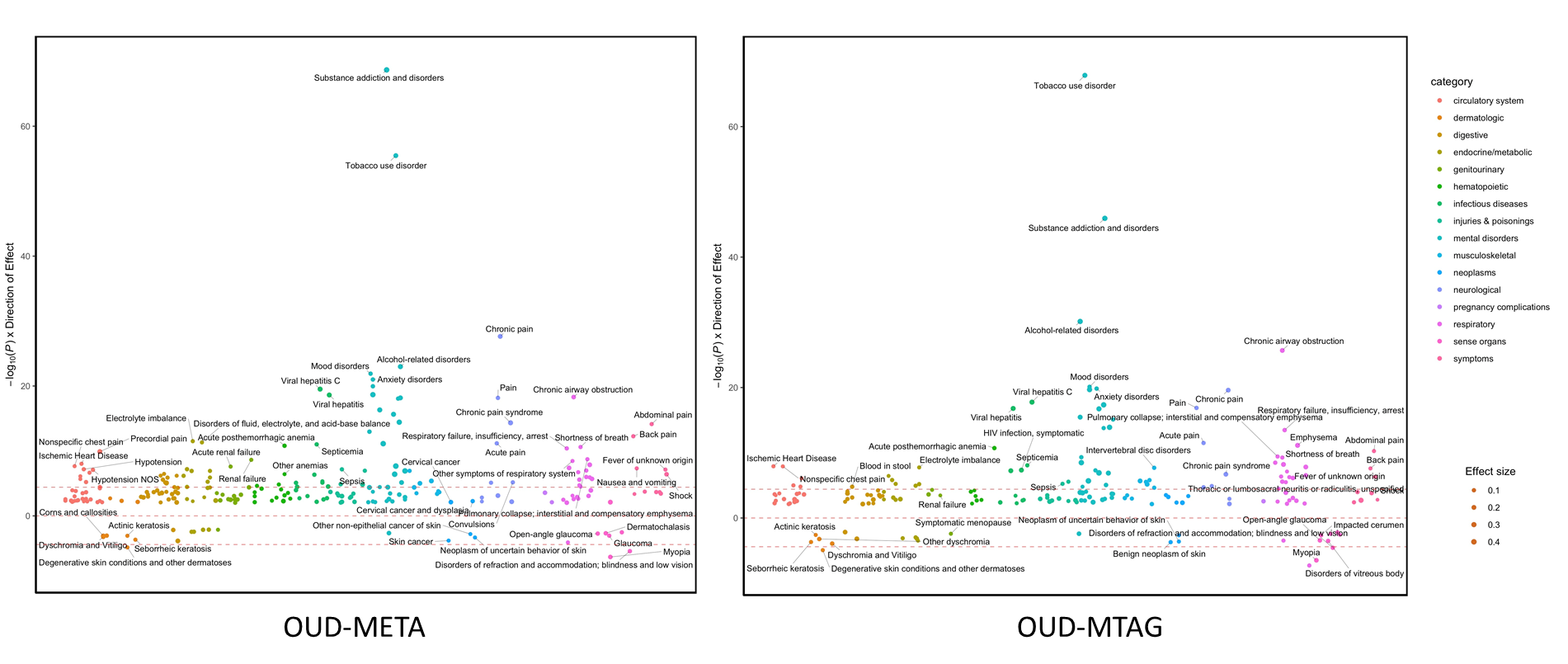
**

***Note.*** Phenome-wide association study (PheWAS) results for 1291 clinical outcomes in BioVU. Y-axis represents the -log_10_(*p-*value) multiplied by the direction of effect. Diameter of data point corresponds to magnitude of effect size (i.e., larger dot=larger effect size). Data points below and above lower and upper red dashed line, respectively, indicate significant PheWAS association exceeding Bonferroni correction (*p*=3.87x10^-05^**).**
